# Glucose-transporter 1 (GLUT1) as a prognostic biomarker: evidence from 14,966 human tumors across 134 cancer types

**DOI:** 10.1186/s12885-025-15527-5

**Published:** 2026-01-10

**Authors:** Seyma Büyücek, Katharina Möller, Florian Viehweger, Ria Schlichter, Anne Menz, Andreas M Luebke, Viktor Reiswich, Martina Kluth, Claudia Hube-Magg, Andrea Hinsch, Florian Lutz, Sören Weidemann, Frank Jacobsen, Maximilian Lennartz, David Dum, Christian Bernreuther, Patrick Lebok, Guido Sauter, Andreas H Marx, Ronald Simon, Christoph Fraune, Natalia Gorbokon, Eike Burandt, Sarah Minner, Stefan Steurer, Till S Clauditz, Till Krech, Viktoria Chirico

**Affiliations:** 1 Institute of Pathology, University Medical Center Hamburg-Eppendorf, Martinistr. 52, Hamburg, 20246 Germany; 2 Institute of Pathology, Clinical Center Osnabrueck, Osnabrueck, Germany; 3 Department of Pathology, Academic Hospital Fuerth, Fuerth, Germany

**Keywords:** GLUT1, Tissue microarray, Immunohistochemistry, Human cancer, Prognosis

## Abstract

**Background:**

Glucose transporter 1 (GLUT1) is a key protein for transmembranous glucose uptake of cells which is often overexpressed in cancer.

**Methods:**

To better comprehend the prevalence and role of GLUT1 expression in different cancer types, a tissue microarray containing 14,966 samples from 134 different tumor entities was analyzed in this study. GLUT1 expression was generally markedly higher in cancer than in corresponding normal tissues.

**Results:**

A total of 122 of 134 tumor categories showed GLUT1 expression in at least 1 case, and 97 tumor categories included at least 1 case with strong GLUT1 staining. The frequency of GLUT1 positivity was particularly high in squamous cell carcinomas of various sites of origin (92.9%-100%), urothelial neoplasms (91.3%-98.7%), and carcinomas of the ovary and the endometrium (up to 100%). Elevated levels of GLUT1 staining were often associated with parameters of cancer aggressiveness. For example, high GLUT1 staining was associated with invasive growth of urothelial carcinomas (*p* = 0.0003), high grade (*p* = 0.0075), advanced pT stage (*p* = 0.025), and shorter survival (*p* = 0.0031) in clear cell renal cell carcinoma, high grade (*p* = 0.0500) and distant metastasis (*p* = 0.011), as well as shorter recurrence free survival (*p* = 0.0253) in papillary renal cell carcinoma, high grade in invasive breast cancer of no special type (*p* = 0.0003), advanced pT stage (*p* = 0.0008), nodal metastasis (*p* = 0.0007), V1 status (*p* = 0.004), and L1 status (*p* = 0.0008) in colorectal adenocarcinoma, and advanced pT stage (*p* = 0.0459) and MMR deficiency (*p* = 0.0018) in gastric cancer.

**Conclusions:**

It is concluded, that GLUT1 is highly expressed in a broad range of tumor entities, that it occurs more often in malignant than in benign tumors, and that it is linked to aggressive behavior in many different entities. These findings identify GLUT1 as a critical prognostic cancer marker, which may be clinically useful in many cancer types.

**Supplementary Information:**

The online version contains supplementary material available at 10.1186/s12885-025-15527-5.

## Introduction

A metabolic shift from oxidative phosphorylation to aerobic glycolysis occurs frequently in rapidly dividing cancer cells [1]. In order to ascertain the necessary uptake of glucose, cancer cells must increase the production of membrane-bound glucose transport molecules in case of increased aerobic glycolysis. The facilitative glucose transporter family (GLUT) – one of two hexose transporter protein families – molecules are often activated in cancer cells [2]. In particular, GLUT1 – an insulin independent glucose transporter for basal glucose uptake - has been found to be often overexpressed in cancer [3].

**Fig. 1:**
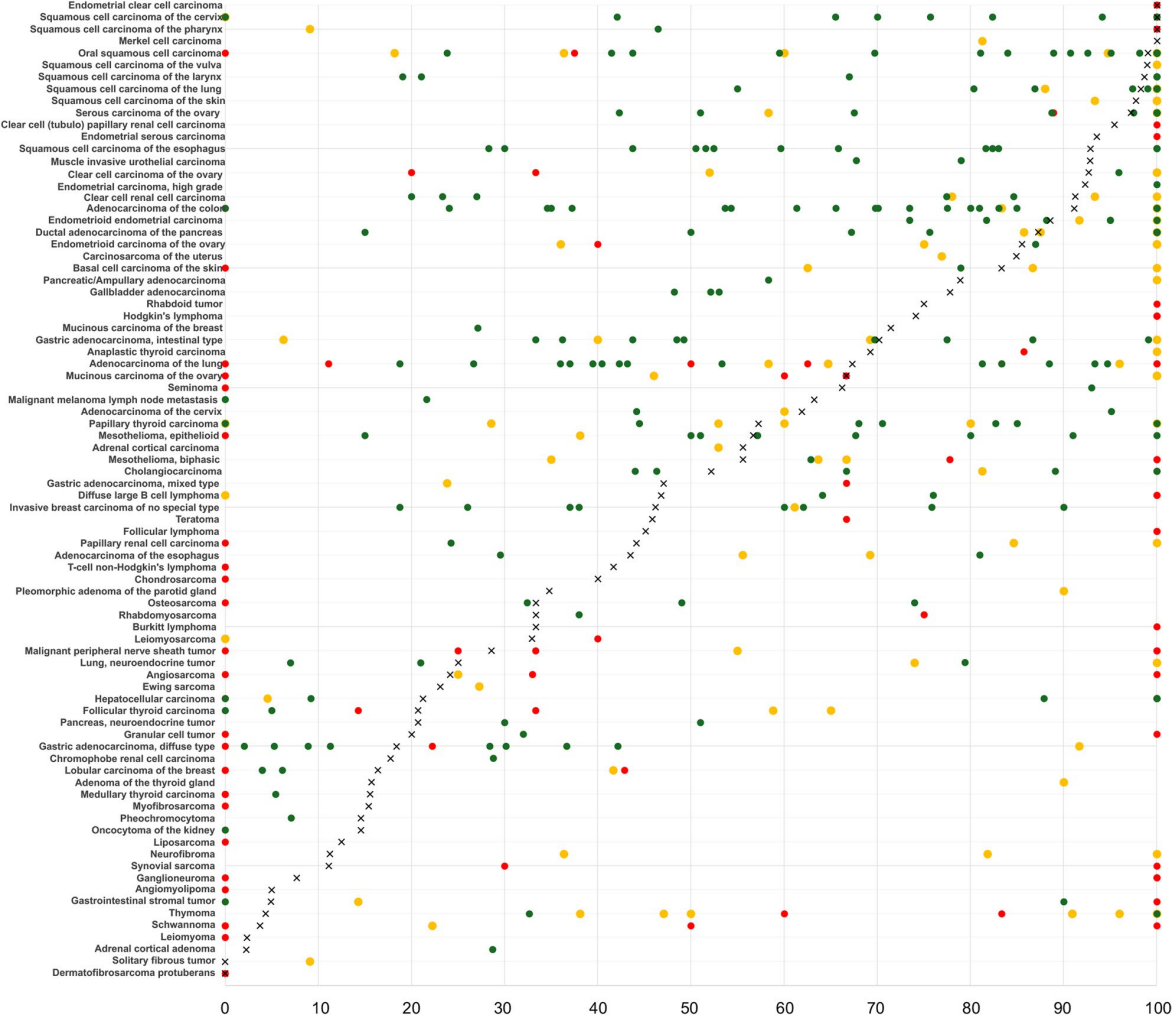
Comparison of GLUT1 immunostaining results with previous GLUT1 studies. An „X“ indicates the fraction of GLUT1 positive cancer cells in the present study, dots indicate the reported frequencies from the literature for comparison: red dots mark studies with ≤10 analyzed tumors, yellow dots mark studies with ≥11 ≤ 25 analyzed tumors and green dots mark studies with > 25 analyzed tumors. All studies are listed in supplementary table 1. Locations of specific tumors types include seminoma of the testis, soft tissue rhabdoid tu-mors, teratomas of the testis, and typical as well as typical and atypical neuroendocrine of the lungs

The markedly higher expression of GLUT1 in many cancers as compared to normal tissues makes GLUT1 immunohistochemistry (IHC) a potential tool for the distinction of malignant from benign cells [3]. GLUT1 expression analysis may serve as a marker for dismal cancer prognosis. Studies have indicated that a high level of GLUT1 expression may be linked to tumor aggressiveness and prognosis in several tumor entities [4–12], Moreover, considering its membranous location, GLUT1 may represent a suitable drug target. Selective GLUT1 inhibitors have been shown to exert cell growth inhibiting effects in in vitro and in vivo model systems – especially of rapidly growing tumor cells [13]. The expression of GLUT1 has already been investigated in numerous IHC studies in various tumor entities. However, the results are profoundly variable. For example, the rate of GLUT1 positivity in the literature ranges from 0% to 100% in pulmonary, 0% to 100% in colorectal, 30% to 81% in esophageal, 44% to 95% in cervical, and 15% to 100% in pancreatic adenocarcinomas, 19% to 90% in invasive breast carcinomas of no special type, 42% to 100% in serous high-grade carcinomas of the ovary, 0% to 100% in angiosarcomas, 35% to 100% in biphasic mesothelioma, and 0% to 100% in diffuse large B cell lymphoma (Fig. 1, Supplementary Table 1). These conflicting data are most probably caused by the use of different antibodies, immunostaining protocols, and criteria to determine GLUT1 positivity in these studies.

To better understand the prevalence and significance of GLUT1 expression in cancer, a comprehensive study analyzing a large number of neoplastic and non-neoplastic tissues under highly standardized conditions is desirable. Therefore, GLUT1 expression was analyzed in more than 14,000 tumor tissue samples from 134 different tumor types and subtypes as well as 76 normal tissue categories by IHC in a tissue microarray (TMA) format in this study.

## Materials and methods

### Tissue microarrays (TMAs)

Our normal tissue TMA was composed of 8 samples each from 8 different donors for each of 76 different normal tissue types (608 samples on one slide). The eight samples per tissue type ensure that there is sufficient analyzable material to assess the GLUT1 status of each organ. The cancer TMAs contained a total of 14,966 primary tumors from 134 tumor types and subtypes, which were selected for sufficient tumor size to allow for TMA construction. Detailed histopathological data were available for cancers of the kidney (*n* = 1757), urinary bladder (*n* = 829), colorectum (*n* = 2351), pancreas (*n* = 598), breast (*n* = 600), stomach (*n* = 327), ovary (*n* = 524), endometrium (*n* = 259), testis (*n* = 565), and the thyroid (*n* = 518). Clinical follow up data were accessible from 850 renal cell cancer patients with a median follow-up time of 39 months. Further details on available general patient data are summarized in Supplementary Table 2. The composition of both normal and cancer TMAs is described in detail in the results section. All samples were from the archives of the Institute of Pathology, University Medical Center Hamburg-Eppendorf, Germany, the Institute of Pathology, Clinical Center Osnabrueck, Germany, and Department of Pathology, Academic Hospital Fuerth, Germany. Tissues were fixed in 4% buffered formalin and then embedded in paraffin. The TMA manufacturing process was described earlier in detail [14, 15]. In brief, one tissue spot (diameter: 0.6 mm) per patient was used. The use of archived remnants of diagnostic tissues for manufacturing of TMAs and their analysis for research purposes as well as patient data analysis has been approved by local laws (HmbKHG, § 12) and by the local ethics committee (Ethics commission Hamburg, WF-049/09). All work has been carried out in compliance with the Helsinki Declaration.

### Immunohistochemistry (IHC)

Freshly cut TMA sections were immunostained on one day and in one experiment. Slides were deparaffinized with xylol, rehydrated through a graded alcohol series and exposed to heat-induced antigen retrieval for 5 min in an autoclave at 121 °C in pH 7.8 Tris-EDTA-Citrat (TEC) puffer. Endogenous peroxidase activity was blocked with Dako REAL Peroxidase-Blocking Solution (Agilent Technologies, Santa Clara, CA, USA; #S2023) for 10 min. Primary antibody specific for GLUT1 (recombinant rabbit monoclonal, MSVA-401R, MS Validated Antibodies, Hamburg, Germany; #4457-401R) was applied at 37 °C for 60 min at a dilution of 1:150. Bound antibody was then visualized using the Dako REAL EnVision Detection System Peroxidase/DAB+, Rabbit/Mouse kit (Agilent Technologies, Santa Clara, CA, USA; #K5007) according to the manufacturer’s directions. The sections were counterstained with hemalaun. All stained tumor spots were manually scored by an experienced pathologist using a microscope. For tumor tissues, the percentage of positive neoplastic cells was estimated, and the staining intensity was semi-quantitatively recorded (0, 1+, 2+, 3+). For statistical analyses, the staining results were categorized into four groups. Tumors without any staining were considered negative. Tumors with 1 + staining intensity in ≤ 70% of tumor cells or 2 + intensity in ≤ 30% of tumor cells were considered weakly positive. Tumors with 1 + staining intensity in > 70% of tumor cells, 2 + intensity in 31–70%, or 3 + intensity in ≤ 30% of tumor cells were considered moderately positive. Tumors with 2 + intensity in > 70% or 3 + intensity in > 30% of tumor cells were considered strongly positive. For survival analysis, tumors were grouped in GLUT1 “low”, including negative, weak and moderate staining, and GLUT1 “high” (strong) staining. For the purpose of antibody validation, the normal tissue TMA was also analyzed by the rabbit polyclonal GLUT1 antibody 355 A-15 (Cell Marque, Rocklin, CA, pH 6.0, 1:200) on a DAKO autostainer Link48 according to a protocol suggested by Agilent DAKO.

### Statistics

Statistical calculations were performed with JMP17^®^ software (SAS^®^, Cary, NC, USA). Contingency tables and the chi²-test were performed to search for associations between GLUT1 immunostaining and tumor phenotype. Survival curves were calculated according to Kaplan-Meier. The Log-Rank test was applied to detect significant differences between groups. Cox-regression analysis was performed to assess the prognostic value of GLUT1 immunostaining compared to tumor stage and nodal stage in clear cell renal cell carcinomas.

## Results

### Technical issues

A total of 12,345 (82.5%) of 14,966 tumor samples were interpretable in our TMA analysis. Non-interpretable samples demonstrated lack of unequivocal tumor cells or absence of entire tissue spots.

### GLUT1 in normal tissues

**Fig. 2:**
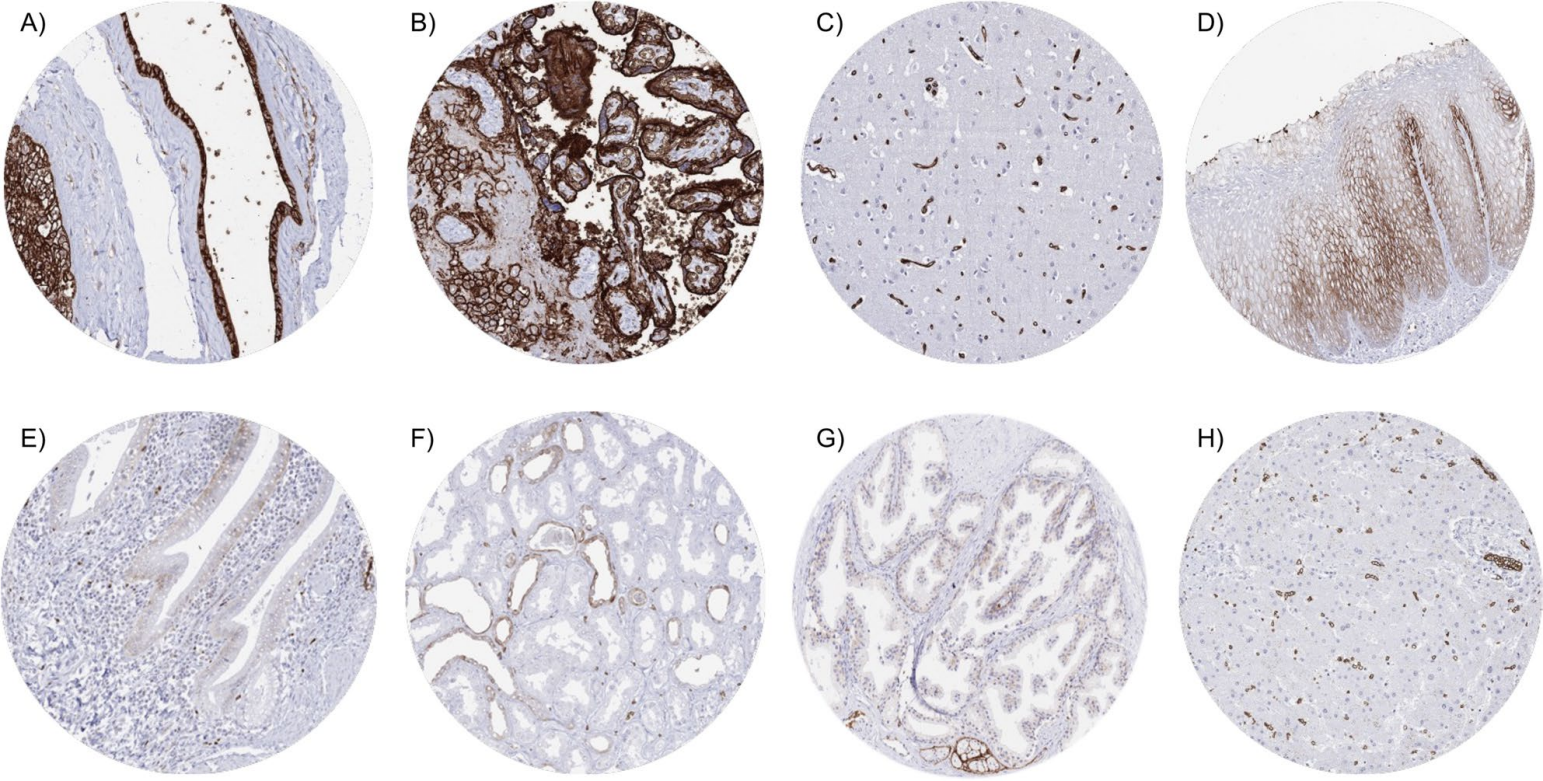
GLUT1 immunostaining in normal tissue. GLUT1 staining was typically membranous but also cytoplasmic. The panels show strong GLUT1 staining in amnion and chorion cells of the placenta (**A**), predominantly membranous staining in cyto- and syncytiotrophoblasts of a mature placenta (**B**), lack of GLUT1 staining of cerebral cells with a strong staining of small vessel cells in the grey cerebrum (**C**), moderate GLUT1 staining of suprabasal cell layers of the anal skin (**D**), focal weak GLUT1 staining in the surface epithelium of the gallbladder (**E**), weak to moderate GLUT1 staining of few collecting ducts in the renal cortex (**F**), weak GLUT1 staining in basal cells of the prostate (**G**), and lack of GLUT1 staining in sinusoidal cells with strong staining of erythrocytes in the liver (**H**)

GLUT1 staining was typically membranous but also cytoplasmic. It was strongest in amnion, chorion, and trophoblast cells of the placenta as well as in erythrocytes and their precursor cells. GLUT1 staining was also moderate to strong in decidua cells. GLUT1 staining of endothelial cells was dependent on the location and tissue type. It ranged from absent to very strong and it was strongest in the brain. A weak to moderate GLUT1 staining was seen in the lower third of squamous epithelium of various sites including skin and tonsil crypts. A weak, preferentially basolateral GLUT1 staining could be seen in the surface epithelium of the stomach. In the intestine and in the gallbladder, epithelial cell staining could focally be observed (often weak). Liver and pancreas were mostly GLUT1 negative, but a focal staining of acinar epithelial cells could be seen in the pancreas. A weak to moderate staining occurred in the urothelium with decreasing intensity from the basal to the superficial cell layers. Few renal tubuli or collecting ducts showed a weak to moderate GLUT1 staining. A faint GLUT1 staining occurred in prostatic basal cells in some samples. Seminal vesicles showed a predominantly basolateral positivity in a fraction of epithelial cells. Acinar cells of the breast, epithelial and stromal cells of the endometrium, as well as fallopian tube epithelium showed a weak GLUT1 staining in some samples. A weak GLUT1 staining occurred in (non-basal) cells of the respiratory epithelium. In tonsil and lymph nodes, a moderate to strong GLUT1 staining of follicular dendritic cells occurred in germinal centers while staining was markedly weaker in some lymphocytic and monocytic interfollicular cells. And a weak to moderate staining was seen in the superficial epithelium of rectum, the follicular dendritic cells of tonsil with dispersed weak staining in the interfollicular cells, the epithelium of seminal vesicle, the epithelium of fallopian tube, decidual cells of the placenta, syncytio- and cytotrophoblast cells of mature placenta, few collecting ducts and renal tubuli of the kidney medulla as well as in basal cell layer of the urothelium of the kidney pelvis. Representative images are shown in Fig. 2. All these cell types were stained by both MSVA-401R and 355 A-15 (Supplementary Fig. 1). Except erythrocyte and endothelial cell staining, GLUT1 staining was absent in muscle, fibrous tissue, fat, brain, non-erythropoietic cells of the bone marrow, salivary glands, Brunner glands, testis, epididymis, ovary, endocervix, lung, adrenal gland, thyroid, parathyroid gland, and the adenohypophysis.

### GLUT1 in tumor tissues

**Fig. 3:**
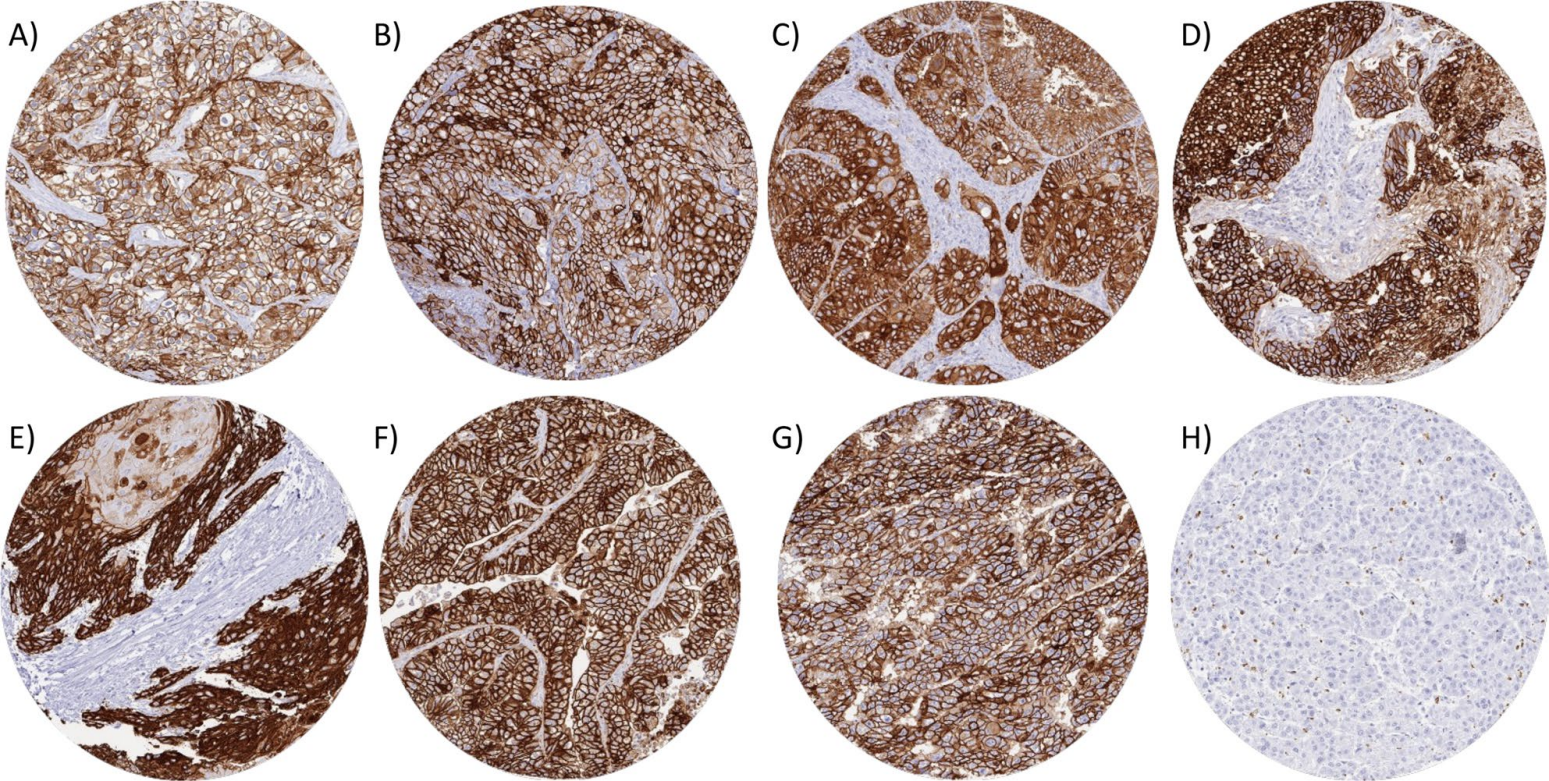
GLUT1 immunostaining in cancer. Strong GLUT1 immunostaining in clear cell renal cell carcinoma (**A**), urothelial carcinoma (**B**), colorectal carcinoma (**C**), serous high-grade ovarian carcinoma (**D**), squamous cell carcinoma of the vulva (**E**), endometrioid endometrial carcinoma (**F**), enbryonel carcinoma of the testis (**G**), and absence of GLUT1 immunostaining in hepatocellular carcinoma (**H**)

**Fig. 4:**
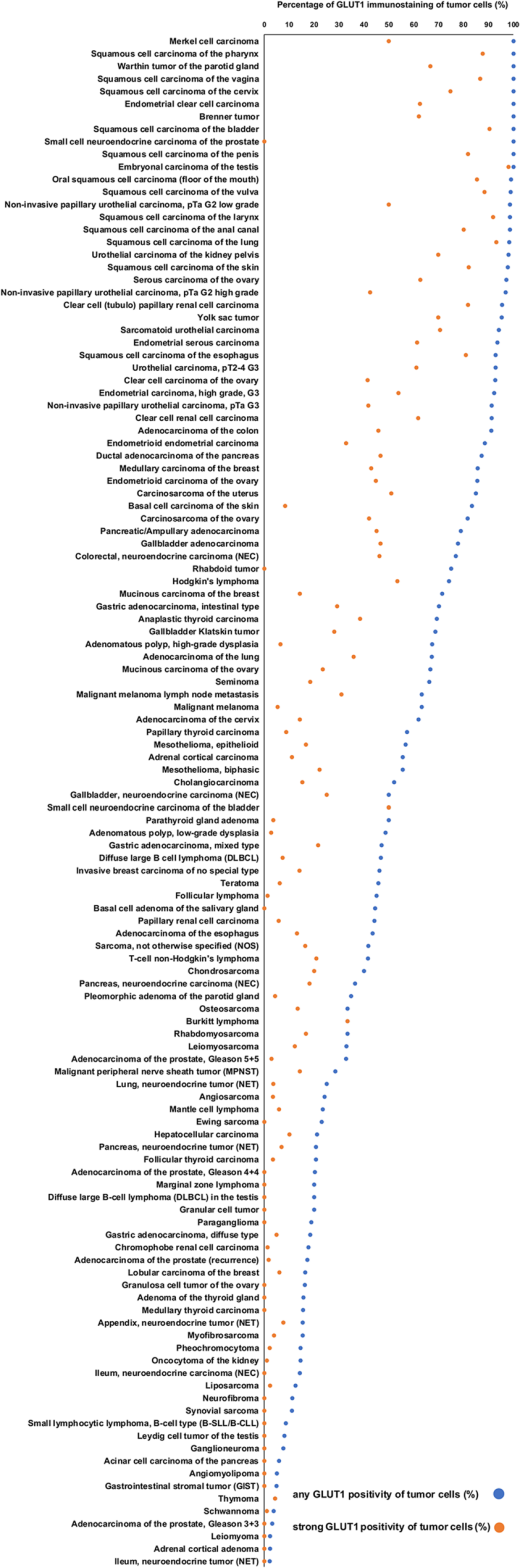
Ranking order of GLUT1 immunostaining in tumors. Both the percentage of positive cases (blue dots) and the percentage of strongly positive cases (orange dots) are shown

**Fig. 5:**
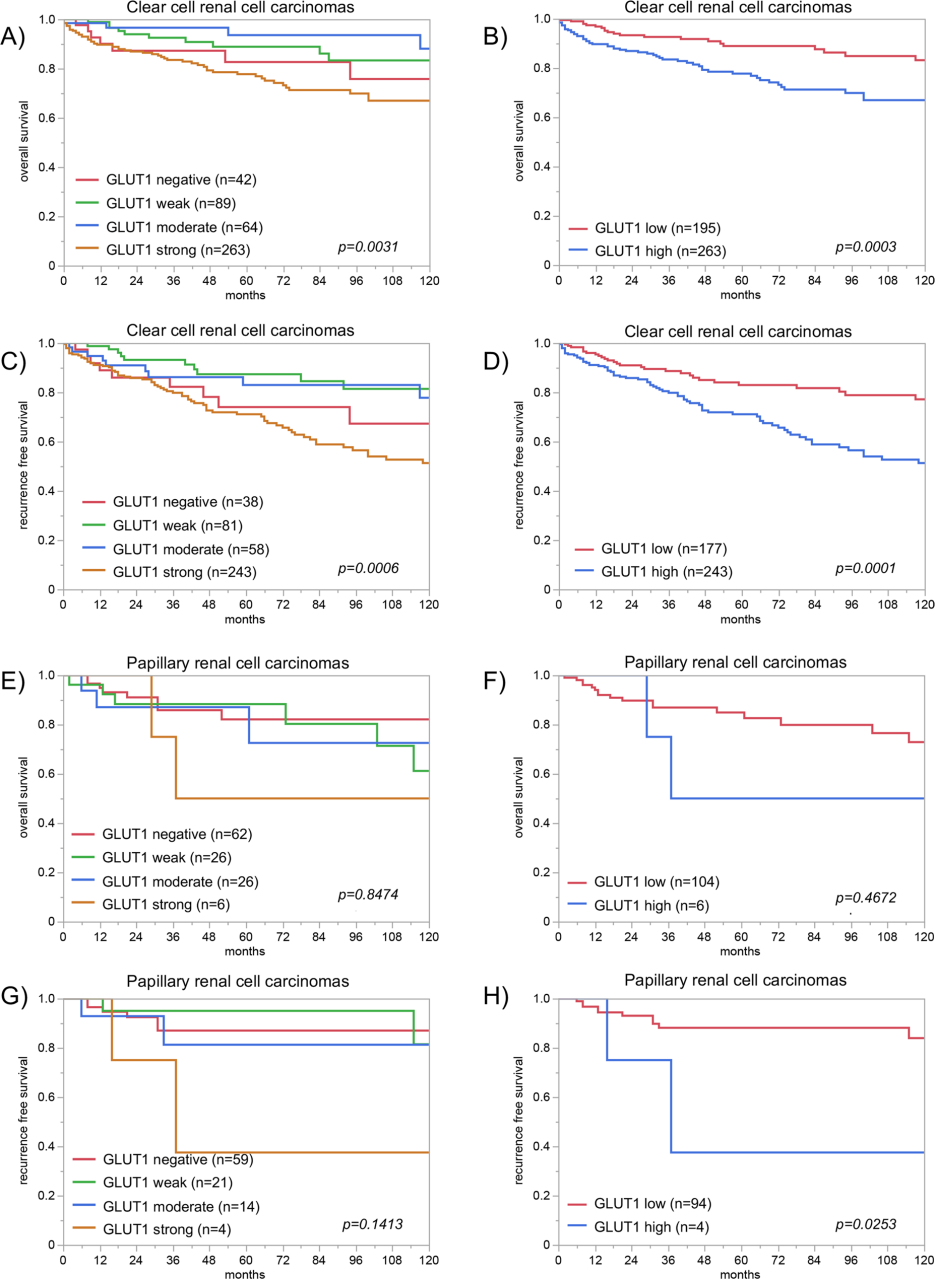
GLUT1 immunostaining and prognosis in (**A**-**D**) clear cell renal cell cancer and in (**E**-**H**) papillary renal cell cancer. **A**, **B**), **E**), and **F**) show overall survival, **C**), **D**), **G**) and **H**) show recurrence free survival. For **B**), **D**), **F** and **H**), tumors were grouped in GLUT1 “low”, including negative, weak and moderate staining, and GLUT1 “high” (strong) staining

GLUT1 immunostaining was generally markedly higher in cancer than in corresponding normal tissues. GLUT1 staining was found in 8208 (66.5%) of the 12,345 interpretable tumor samples, including 2298 (18.6%) with weak, 1615 (13.1%) with moderate, and 4295 (34.8%) with strong positivity. A total of 122 of 134 tumor categories showed GLUT1 expression in at least one case, and 97 tumor categories included at least one case with strong GLUT1 staining (Table 1). The frequency of GLUT1 positivity was particularly high in squamous cell carcinomas of various sites of origin (92.9%-100%), urothelial neoplasms (91.3%-98.7%), carcinomas of the ovary and the endometrium (up to 100%), small cell neuroendocrine carcinomas of the prostate (100%), embryonal carcinomas of the testis (100%), renal cell carcinomas (up to 95.5%), as well as adenocarcinomas of different sites (up to 91.1%). Representative images of GLUT1 positive tumors are shown in Fig. 3. A graphical representation of a ranking order of GLUT1 positive cancers and of strongly positive cancers is given in Fig. 4. Elevated levels of GLUT1 staining were often associated with parameters of cancer aggressiveness in our patients (Table 2). High GLUT1 staining was associated with invasive growth of urothelial carcinomas (*p* = 0.0003 for pTa vs. pT2-4), high ISUP grade (*p* = 0.0075), advanced pT stage (*p* = 0.025), and overall survival (*p* = 0.0031; Figs. 5 A; *p* = 0.0003 5B) as well as recurrence free survival (Figs. 5 C *p* = 0.0006; 5D *p* = 0.0001) in clear cell renal cell carcinomas. High ISUP grade (*p* = 0.0500) and distant metastasis (*p* = 0.011) as well as recurrence free survival (*p* = 0.0253, Fig. 5H) in papillary renal cell carcinomas, high grade in invasive breast carcinomas of no special type (*p* = 0.0003), advanced pT stage (*p* = 0.0008), nodal metastasis (*p* = 0.0007), V1 status (*p* = 0.004), and L1 status (*p* = 0.0008) in colorectal adenocarcinoma, and advanced pT stage (*p* = 0.0459) and MMR deficiency (*p* = 0.0018) in gastric cancer. GLUT1 expression was also more prominent in 129 Gleason 4 + 4 and 5 + 5 cancers (37.1%) than in 62 Gleason 3 + 3 cancers (3.2%, *p* < 0.0001) of the prostate and tended to be more frequent in 31 neuroendocrine cancers (54.8%) than in 293 neuroendocrine tumors (16.0%; *p* = 0.0540) from various organs. In a multivariate analysis including pT and pN in in clear cell renal cell carcinomas, the prognostic value of GLUT1 was not independent from these parameters, neither for recurrence-free survival nor for overall survival (Supplementary Table 3). GLUT1 staining was not significantly related to parameters of cancer aggressiveness in muscle-invasive urothelial carcinoma, serous high-grade carcinoma of the ovary, endometrioid endometrium carcinoma, papillary thyroid cancer, and testicular seminoma (data not shown).

**Table 1:** GLUT1 immunostaining in human tumors

	Tumor entity	on TMA (*n*)	analyzable (*n*)	negative (%)	weak (%)	moderate (%)	strong (%)
Tumors of the skin	Basal cell carcinoma of the skin	41	24	16.7	62.5	12.5	8.3
	Squamous cell carcinoma of the skin	95	89	2.2	4.5	11.2	82.0
	Malignant melanoma	19	19	36.8	47.4	10.5	5.3
	Malignant melanoma lymph node metastasis	86	68	36.8	16.2	16.2	30.9
	Merkel cell carcinoma	2	2	0.0	0.0	50.0	50.0
Tumors of the head and neck	Squamous cell carcinoma of the larynx	109	74	1.4	2.7	4.1	91.9
	Squamous cell carcinoma of the pharynx	60	49	0.0	2.0	10.2	87.8
	Oral squamous cell carcinoma (floor of the mouth)	130	103	1.0	4.9	8.7	85.4
	Pleomorphic adenoma of the parotid gland	50	23	65.2	30.4	0.0	4.3
	Warthin tumor of the parotid gland	49	33	0.0	18.2	15.2	66.7
	Basal cell adenoma of the salivary gland	15	9	55.6	33.3	11.1	0.0
Tumors of the lung, pleura and thymus	Adenocarcinoma of the lung	196	159	32.7	22.0	9.4	35.8
	Squamous cell carcinoma of the lung	80	58	1.7	0.0	5.2	93.1
	Mesothelioma, epithelioid	40	30	43.3	30.0	10.0	16.7
	Mesothelioma, biphasic	29	18	44.4	16.7	16.7	22.2
	Thymoma	29	23	95.7	0.0	0.0	4.3
	Lung, neuroendocrine tumor (NET)	29	28	75.0	17.9	3.6	3.6
Tumors of the female genital tract	Squamous cell carcinoma of the vagina	30	30	0.0	6.7	6.7	86.7
	Squamous cell carcinoma of the vulva	107	95	1.1	0.0	10.5	88.4
	Squamous cell carcinoma of the cervix	88	83	0.0	9.6	15.7	74.7
	Adenocarcinoma of the cervix	23	21	38.1	33.3	14.3	14.3
	Endometrioid endometrial carcinoma	288	271	11.4	24.0	31.7	32.8
	Endometrial serous carcinoma	36	31	6.5	19.4	12.9	61.3
	Carcinosarcoma of the uterus	57	53	15.1	13.2	20.8	50.9
	Endometrial carcinoma, high grade, G3	13	13	7.7	0.0	38.5	53.8
	Endometrial clear cell carcinoma	9	8	0.0	12.5	25.0	62.5
	Endometrioid carcinoma of the ovary	93	76	14.5	19.7	21.1	44.7
	Serous carcinoma of the ovary	530	465	2.8	17.0	17.6	62.6
	Mucinous carcinoma of the ovary	75	51	33.3	27.5	15.7	23.5
	Clear cell carcinoma of the ovary	51	41	7.3	22.0	29.3	41.5
	Carcinosarcoma of the ovary	47	38	18.4	18.4	21.1	42.1
	Granulosa cell tumor of the ovary	44	37	83.8	16.2	0.0	0.0
	Leydig cell tumor of the ovary	4	4	100.0	0.0	0.0	0.0
	Sertoli cell tumor of the ovary	1	1	100.0	0.0	0.0	0.0
	Sertoli Leydig cell tumor of the ovary	3	3	100.0	0.0	0.0	0.0
	Steroid cell tumor of the ovary	3	3	100.0	0.0	0.0	0.0
	Brenner tumor	32	29	0.0	10.3	27.6	62.1
Tumors of the breast	Invasive breast carcinoma of no special type	499	418	53.8	23.0	9.1	14.1
	Lobular carcinoma of the breast	150	116	83.6	6.0	4.3	6.0
	Medullary carcinoma of the breast	8	7	14.3	28.6	14.3	42.9
	Tubular carcinoma of the breast	2	2	100.0	0.0	0.0	0.0
	Mucinous carcinoma of the breast	7	7	28.6	57.1	0.0	14.3
Tumors of the digestive system	Adenomatous polyp, low-grade dysplasia	50	37	51.4	43.2	2.7	2.7
	Adenomatous polyp, high-grade dysplasia	50	46	32.6	50.0	10.9	6.5
	Adenocarcinoma of the colon	2483	2130	8.9	21.2	24.1	45.8
	Gastric adenocarcinoma, diffuse type	215	163	81.6	10.4	3.1	4.9
	Gastric adenocarcinoma, intestinal type	215	161	29.8	31.1	9.9	29.2
	Gastric adenocarcinoma, mixed type	62	51	52.9	17.6	7.8	21.6
	Adenocarcinoma of the esophagus	83	46	56.5	21.7	8.7	13.0
	Squamous cell carcinoma of the esophagus	76	42	7.1	4.8	7.1	81.0
	Squamous cell carcinoma of the anal canal	91	70	1.4	8.6	10.0	80.0
	Cholangiocarcinoma	58	46	47.8	30.4	6.5	15.2
	Gallbladder adenocarcinoma	51	45	22.2	20.0	11.1	46.7
	Gallbladder Klatskin tumor	42	32	31.3	34.4	6.3	28.1
	Hepatocellular carcinoma	312	297	78.8	7.4	3.7	10.1
	Ductal adenocarcinoma of the pancreas	659	463	12.7	27.2	13.4	46.7
	Pancreatic/Ampullary adenocarcinoma	98	71	21.1	23.9	9.9	45.1
	Acinar cell carcinoma of the pancreas	18	17	94.1	5.9	0.0	0.0
	Gastrointestinal stromal tumor (GIST)	62	61	95.1	3.3	1.6	0.0
	Appendix, neuroendocrine tumor (NET)	25	13	84.6	7.7	0.0	7.7
	Colorectal, neuroendocrine tumor (NET)	12	9	100.0	0.0	0.0	0.0
	Ileum, neuroendocrine tumor (NET)	53	47	97.9	2.1	0.0	0.0
	Pancreas, neuroendocrine tumor (NET)	101	87	79.3	10.3	3.4	6.9
	Colorectal, neuroendocrine carcinoma (NEC)	14	13	23.1	15.4	15.4	46.2
	Ileum, neuroendocrine carcinoma (NEC)	8	7	85.7	0.0	14.3	0.0
	Gallbladder, neuroendocrine carcinoma (NEC)	4	4	50.0	25.0	0.0	25.0
	Pancreas, neuroendocrine carcinoma (NEC)	14	11	63.6	18.2	0.0	18.2
Tumors of the urinary system	Non-invasive papillary urothelial ca., G2 low grade	87	76	1.3	23.7	25.0	50.0
	Non-invasive papillary urothelial ca., G2 high grade	80	66	3.0	27.3	27.3	42.4
	Non-invasive papillary urothelial carcinoma, pTa G3	126	103	8.7	35.0	14.6	41.7
	Urothelial carcinoma, pT2-4 G3	735	488	7.2	18.6	13.1	61.1
	Squamous cell carcinoma of the bladder	22	21	0.0	4.8	4.8	90.5
	Small cell neuroendocrine carcinoma of the bladder	5	4	50.0	0.0	0.0	50.0
	Sarcomatoid urothelial carcinoma	25	17	5.9	23.5	0.0	70.6
	Urothelial carcinoma of the kidney pelvis	62	53	1.9	15.1	13.2	69.8
	Clear cell renal cell carcinoma	1287	1143	8.7	16.8	12.7	61.8
	Papillary renal cell carcinoma	368	315	55.9	25.4	13.0	5.7
	Clear cell (tubulo) papillary renal cell carcinoma	26	22	4.5	9.1	4.5	81.8
	Chromophobe renal cell carcinoma	170	152	82.2	10.5	5.9	1.3
	Oncocytoma of the kidney	257	199	85.4	10.6	3.0	1.0
Tumors of the male genital organs	Adenocarcinoma of the prostate, Gleason 3 + 3	83	62	96.8	3.2	0.0	0.0
	Adenocarcinoma of the prostate, Gleason 4 + 4	80	59	79.7	16.9	3.4	0.0
	Adenocarcinoma of the prostate, Gleason 5 + 5	85	70	67.1	22.9	7.1	2.9
	Adenocarcinoma of the prostate (recurrence)	258	237	82.7	12.2	3.4	1.7
	Small cell neuroendocrine carcinoma of the prostate	2	1	0.0	100.0	0.0	0.0
	Seminoma	682	598	33.8	30.3	17.6	18.4
	Embryonal carcinoma of the testis	54	51	0.0	0.0	2.0	98.0
	Leydig cell tumor of the testis	31	25	92.0	4.0	4.0	0.0
	Sertoli cell tumor of the testis	2	2	100.0	0.0	0.0	0.0
	Sex cord stromal tumor of the testis	1	1	100.0	0.0	0.0	0.0
	Spermatocytic tumor of the testis	1	1	100.0	0.0	0.0	0.0
	Yolk sac tumor	53	43	4.7	16.3	9.3	69.8
	Teratoma	53	48	54.2	20.8	18.8	6.3
	Squamous cell carcinoma of the penis	92	77	0.0	6.5	11.7	81.8
Tumors of endocrine organs	Adenoma of the thyroid gland	63	51	84.3	13.7	2.0	0.0
	Papillary thyroid carcinoma	341	262	42.7	36.3	12.2	8.8
	Follicular thyroid carcinoma	109	58	79.3	12.1	5.2	3.4
	Medullary thyroid carcinoma	57	45	84.4	11.1	4.4	0.0
	Parathyroid gland adenoma	43	28	50.0	32.1	14.3	3.6
	Anaplastic thyroid carcinoma	19	13	30.8	23.1	7.7	38.5
	Adrenal cortical adenoma	48	44	97.7	2.3	0.0	0.0
	Adrenal cortical carcinoma	27	27	44.4	40.7	3.7	11.1
	Pheochromocytoma	51	48	85.4	12.5	0.0	2.1
Tumors of hematopoetic and lymphoid tissues	Hodgkin’s lymphoma	103	58	25.9	5.2	15.5	53.4
	Small lymphocytic lymphoma, B-cell type	50	46	91.3	8.7	0.0	0.0
	Diffuse large B cell lymphoma	113	109	53.2	30.3	9.2	7.3
	Follicular lymphoma	88	82	54.9	36.6	7.3	1.2
	T-cell non-Hodgkin’s lymphoma	25	24	58.3	8.3	12.5	20.8
	Mantle cell lymphoma	18	17	76.5	17.6	0.0	5.9
	Marginal zone lymphoma	16	15	80.0	13.3	6.7	0.0
	Diffuse large B-cell lymphoma in the testis	16	15	80.0	13.3	6.7	0.0
	Burkitt lymphoma	5	3	66.7	0.0	0.0	33.3
Tumors of soft tissue and bone	Granular cell tumor	23	20	80.0	20.0	0.0	0.0
	Leiomyoma	50	43	97.7	0.0	2.3	0.0
	Leiomyosarcoma	94	82	67.1	18.3	2.4	12.2
	Liposarcoma	96	88	87.5	6.8	3.4	2.3
	Malignant peripheral nerve sheath tumor	15	14	71.4	14.3	0.0	14.3
	Myofibrosarcoma	26	26	84.6	11.5	0.0	3.8
	Angiosarcoma	42	29	75.9	10.3	10.3	3.4
	Angiomyolipoma	91	80	95.0	3.8	1.3	0.0
	Dermatofibrosarcoma protuberans	21	14	100.0	0.0	0.0	0.0
	Ganglioneuroma	14	13	92.3	0.0	7.7	0.0
	Kaposi sarcoma	8	3	100.0	0.0	0.0	0.0
	Neurofibroma	117	89	88.8	7.9	3.4	0.0
	Sarcoma, not otherwise specified	74	67	58.2	20.9	4.5	16.4
	Paraganglioma	41	37	81.1	16.2	2.7	0.0
	Ewing sarcoma	23	13	76.9	15.4	7.7	0.0
	Rhabdomyosarcoma	7	6	66.7	16.7	0.0	16.7
	Schwannoma	122	107	96.3	1.9	0.9	0.9
	Synovial sarcoma	12	9	88.9	0.0	11.1	0.0
	Osteosarcoma	19	15	66.7	0.0	20.0	13.3
	Chondrosarcoma	15	10	60.0	10.0	10.0	20.0
	Rhabdoid tumor	5	4	25.0	25.0	50.0	0.0
	Solitary fibrous tumor	17	16	100.0	0.0	0.0	0.0

**Table 2:** GLUT1 immunostaining and tumor phenotype

		*n*	GLUT1 immunostaining				
			negative (%)	weak (%)	moderate (%)	strong (%)	P**
Invasive breast carcinoma of no special type	pT1	147	60.5	21.8	6.1	11.6	*0.1703*
	pT2	174	48.3	26.4	10.3	14.9	
	pT3-4	34	50.0	14.7	11.8	23.5	
	G1	13	76.9	15.4	0.0	7.7	*0.0003*
	G2	194	61.9	25.3	6.2	6.7	
	G3	151	39.1	23.2	12.6	25.2	
	pN0	180	51.7	25.6	7.8	15.0	*0.6222*
	pN+	143	57.3	20.3	9.1	13.3	
Clear cell renal cell carcinoma	ISUP 1	256	7.8	17.2	14.5	60.5	*0.0075*
	ISUP 2	384	6.5	14.8	13.0	65.6	
	ISUP 3	255	14.5	18.4	12.2	54.9	
	ISUP 4	71	4.2	9.9	5.6	80.3	
	UICC 1	299	8.0	23.1	14.0	54.8	*0.3072*
	UICC 2	36	16.7	22.2	13.9	47.2	
	UICC 3	88	8.0	17.0	10.2	64.8	
	UICC 4	68	10.3	11.8	11.8	66.2	
	pT1	629	7.6	19.9	13.4	59.1	*0.025*
	pT2	131	8.4	18.3	9.2	64.1	
	pT3-4	325	9.8	10.5	11.4	68.3	
	pN0	169	7.1	16.0	10.7	66.3	*0.8915*
	pN+	25	4.0	16.0	8.0	72.0	
	pM0	104	6.7	13.5	16.3	63.5	*0.8024*
	pM+	89	7.9	11.2	12.4	68.5	
papillary renal cell carcinoma	ISUP 1	37	56.8	29.7	10.8	2.7	*0.0500*
	ISUP 2	125	58.4	26.4	10.4	4.8	
	ISUP 3	73	37.0	32.9	21.9	8.2	
	ISUP 4	6	50.0	16.7	0.0	33.3	
	UICC 1	91	53.8	28.6	16.5	1.1	*0.0913*
	UICC 2	12	66.7	8.3	8.3	16.7	
	UICC 3	5	80.0	20.0	0.0	0.0	
	UICC 4	12	41.7	16.7	25.0	16.7	
	pT1	191	53.9	30.4	11.5	4.2	*0.23*
	pT2	47	55.3	21.3	17.0	6.4	
	pT3-4	32	50.0	12.5	18.8	18.8	
	pN0	25	56.0	24.0	20.0	0.0	*0.23*
	pN+	14	35.7	7.1	21.4	35.7	
	pM0	26	57.7	34.6	7.7	0.0	*0.011*
	pM+	12	33.3	8.3	25.0	33.3	
Urothelial bladder carcinomas	pTa G2 low	76	1.3	23.7	25.0	50.0	*0.0596*
	pTa G2 high	66	3.0	27.3	27.3	42.4	
	pTa G3	80	10.0	38.8	12.5	38.8	
	pT2	85	5.9	17.6	14.1	62.4	*0.5274*
	pT3	178	5.6	20.8	12.4	61.2	
	pT4	83	13.3	18.1	10.8	57.8	
	G2	18	11.1	27.8	16.7	44.4	*0.5603
	G3	328	7.3	18.9	12.2	61.6	
	pN0	197	7.1	17.3	14.7	60.9	*0.3883
	pN+	132	7.6	22.0	9.1	61.4	
Adenocarcinoma of the stomach	pT1-2	45	66.7	13.3	4.4	15.6	*0.0459*
	pT3	106	46.2	26.4	8.5	18.9	
	pT4	108	57.4	10.2	4.6	27.8	
	pN0	64	56.3	20.3	3.1	20.3	*0.4804*
	pN+	195	52.8	16.4	7.7	23.1	
	MMR proficient	224	51.3	18.8	7.6	22.3	*0.0018*
	MMR deficient	31	16.1	25.8	6.5	51.6	
Adenocarcinoma of the colon	pT1	77	13.0	31.2	27.3	28.6	*0.0008*
	pT2	389	7.2	25.4	29.0	38.3	
	pT3	1148	8.5	20.7	24.1	46.6	
	pT4	406	9.9	16.5	20.2	53.4	
	pN0	1042	9.0	24.1	27.1	39.8	*0.0007*
	pN+	972	8.3	17.8	21.8	52.1	
	V0	1450	8.3	22.8	25.6	43.3	*0.004*
	V1	530	9.4	17.0	20.9	52.6	
	L0	635	9.9	23.9	28.0	38.1	*0.0008*
	L1	1353	8.1	20.1	22.6	49.2	
	right side	412	8.3	19.9	24.3	47.6	*0.7117*
	left side	1117	9.6	21.1	24.6	44.7	
	MMR proficient	1029	7.3	20.4	25.7	46.6	*0.3024*
	MMR deficient	76	15.8	26.3	21.1	36.8	
	RAS wildtype	458	10.9	23.4	24.2	41.5	*0.1341*
	RAS mutation	346	8.1	18.5	26.6	46.8	
	BRAF wildtype	124	11.3	25.8	29.0	33.9	*0.1732*
	BRAF V600E mutation	22	13.6	27.3	9.1	50.0	
Adenocarcinoma of the pancreas	pT1	8	25.0	50.0	12.5	12.5	*0.0544*
	pT2	50	16.0	30.0	6.0	48.0	
	pT3	279	11.8	25.8	14.3	48.0	
	pT4	18	33.3	0.0	22.2	44.4	
	G1	11	18.2	9.1	9.1	63.6	*0.7330*
	G2	254	13.0	28.0	13.4	45.7	
	G3	74	12.2	23.0	14.9	50.0	
	pN0	70	7.1	35.7	12.9	44.3	*0.0863*
	pN+	284	15.5	23.2	13.7	47.5	
	R0	178	16.9	30.3	13.5	39.3	*0.2864*
	R1	150	11.3	20.7	14.0	54.0	
	MMR proficient	322	14.3	25.5	13.4	46.9	*0.6886*
	MMR deficient	2	0.0	50.0	0.0	50.0	
Endometrioid carcinoma of the ovary	pT1	25	20.0	32.0	16.0	32.0	*0.2248*
	pT2	5	20.0	0.0	40.0	40.0	
	pT3	6	16.7	0.0	16.7	66.7	
	pN0	23	26.1	30.4	17.4	26.1	*0.1088*
	pN1	8	0.0	0.0	37.5	62.5	

*abbreviation*: *pT* pathological tumor stage, *G* Grade, *pN* pathological lymph node status, *pM* pathological status of distant metastasis, *V* venous invasion, *L* lymphatic invasion, *MMR* mismatch repair, *ISUP* international socity of urological Pathology, *UICC* union for internatinal cancer Control

*only in pT2-4 urothelial bladder carcinomas

**after bonferroni correction

## Discussion

Our successful analysis of 12,345 tumors from 134 different tumor categories provides a comprehensive overview on GLUT1 expression in cancer. That GLUT1 immunostaining was generally more intense in tumors than in normal tissues and that GLUT1 expression and even high-level GLUT1 expression occurred in a variable fraction of almost all tumor categories is reflective of the well-known phenomenon, that dividing tumor cells tend to increase their glucose consumption and lactate production by aerobic glycolysis [1]. An increased GLUT1 expression results in an increased glucose uptake which can either occur to ensure tumor cell survival in a nutrient deprived environment or to further accelerate the metabolism in the presence of physiological oxygen concentrations and functional mitochondria [2, 3].

The ranking order of neoplasms according to the rate of GLUT1 positivity is the main result of this study. Although more than 500 previous studies have employed IHC to analyze GLUT1 expression in cancers, this information could hardly be compiled from the literature due to the high variability of previous data (summarized in Fig. 1). Our data show that squamous cell carcinomas of various sites of origin, urothelial neoplasms, carcinomas of the ovary and the endometrium, rapidly proliferating tumors such as small cell carcinomas or embryonal carcinomas of the testis, renal cell carcinomas, as well as adenocarcinomas of the colorectum, the pancreas, stomach, the lung, and the biliary tract were among the most commonly GLUT1 positive cancers and showed the highest levels of expression. These cancers might be the best candidates for treatment with GLUT1 inhibitors several of which have demonstrated efficacy in preclinical studies, particularly in models of fast-growing tumor cells with high glucose consumption. For example, the specific GLUT1 inhibitor BAY-876 impaired the growth of triple negative breast cancer cells in vitro [16] and in mouse models [17]. The GLUT1 and NAMPT inhibitor STF-31 and its derivates, STF-62,247 and STF-118,804, inhibited proliferation and survival of cultured cells of neuroendocrine tumors [18] and head and neck squamous cell carcinomas [19], reduced the stem-like properties of pancreatic cancer cells [20], sensitized multiple myeloma cells to chemotherapeutic reagents [21], and selectively killed renal cell carcinoma cells [22]. WZB117, another inhibitor of GLUT1 and related proteins, can resensitize breast cancer cells to radiation [23] or chemotherapy [24], overcome imatinib resistance in gastrointestinal stromal tumor cells [25], and enhance the anti-tumor effect of the VEGFR inhibitor apatinib in malignant melanoma cells [26]. However, no GLUT1-based therapy has yet been successfully transferred into clinical practice, as the almost ubiquitous expression of some levels of GLUT1 in normal tissue poses a challenge for potential GLUT1 inhibitors. It will be interesting to see whether the use of nanomaterials that deliver active substances directly and specifically to tumor cells could represent a breakthrough in GLUT1-targeted therapies [27].

Given the abundancy of GLUT1 expression in all types of cancer, GLUT1 IHC is obviously not well suited for the distinction of different tumor types in surgical pathology. However, due to its predominant expression in neoplastic as compared to non-neoplastic tissues, GLUT1 positivity may argue for a neoplastic process in several types of biopsies. For example, GLUT1 IHC is being used as a diagnostic tool in cutaneous vascular lesions where GLUT1 IHC is positive in many hemangiomas and especially in infantile hemangioma while it is negative in vascular malformations [28]. Although significant GLUT1 staining occurs in several benign tumors, GLUT1 positivity may serve as a marker for malignancy in specific diagnostic settings. For example, the GLUT1 positivity rate was markedly higher in malignant peripheral nerve sheet tumors (28.6%) than in schwannomas (3.7%), in leiomyosarcomas (32.9) than in leiomyomas (2.3%), in Warthin tumors of the parotid gland (100%) than in pleomorphic adenoma (52.2%), in colon adenocarcinomas (96.8%) than in high-grade dysplasia (78.3%), or in papillary thyroid carcinoma (64.6%) than in thyroid adenoma (15.7%).

The availability of several large cohorts of tumors of the same histological type enabled us to analyze the relationship between GLUT1 expression and parameters of cancer aggressiveness. These data identify GLUT1 expression as a striking prognostic marker in many different cancer entities. Significant associations between high GLUT1 expression and key features of cancer aggressiveness such as high grade and poor prognosis in renal cell carcinomas, advanced pT stage and nodal metastasis in colorectal cancer, invasive tumor growth in urothelial carcinoma, high grade in breast cancer, and advanced pT stage in gastric adenocarcinoma fit well with the known role of GLUT1 overexpression to provide tumor cells a growth advantage by enabling an optimized uptake of available glucose molecules [2, 3]. Our data are also in line with various earlier studies suggesting a striking prognostic role of GLUT1 overexpression in additional cancer types [4–12, 29–32] although other studies have failed to determine a prognostic relevance of GLUT1 expression [33–36]. The significant role of GLUT1 overexpression for tumor development and progression is also underscored by the increasing rate of GLUT1 positivity from normal to benign neoplasms, malignant neoplasms, and aggressive malignant neoplasms as seen in many entities in our cohort. In their entirety, our data are consistent with a role of GLUT1 as a master prognostic feature in cancer, and this observation is also consistent with its pivotal biologic role.

It is a limitation of our study that some tumor types without detectable GLUT1 staining are represented by fewer than 20 samples. It cannot be ruled out that a certain degree of positivity would have been detected if more samples had been analyzed. However, the use of TMAs is an advantage of our study. The approach of using a 0.6 mm core per tumor maximizes standardization, as the same amount of tissue (approximately 0.28 mm²) is evaluated for each tumor. In contrast, the use of multiple cores per tumor tends to lead to statistical bias, as most samples from a tumor are not interpretable. Both external studies and our own work show that a single 0.6 mm core per cancer is sufficient to reveal clinically relevant associations between molecular markers and tumor phenotype [37]. For example, Torhorst et al. [38] examined 1–4 TMA cores per tumor for PR, ER, and p53 and showed that established correlations between these markers and patient prognosis were found regardless of whether three cores were analyzed separately or their results were combined into a single overall value.

Considering the large scale of our study, our assay was extensively validated according to the recommendations of the International Working Group of Antibody Validation (IWGAV) [39] by comparing our IHC findings in normal tissues with data obtained by another independent anti-GLUT1 antibody and RNA data derived from three different publicly accessible databases [40–43]. To ensure an as broad as possible range of proteins to be tested for a possible cross-reactivity, 76 different normal tissue categories were included in this analysis. Validity of our IHC assay was supported by the detection of the strongest GLUT1 positivity by IHC in the tissues with highest GLUT1 RNA levels including placenta, bone marrow and tissues covered by squamous epithelium or urothelium. While significant RNA expression was lacking in other tissues with IHC positive cell types (lymph node, germinal centers of the tonsil, kidney, prostate, seminal vesicle, stomach, rectum, gallbladder, endometrium, fallopian tube, decidua cells, and the respiratory epithelium), these were all confirmed by a staining with the independent polyclonal GLUT1 antibody 355A-15.

In summary, our data show that GLUT1 is highly expressed in a broad range of tumor entities, that it occurs more often in malignant than in benign tumors, and that it is linked to aggressive behavior in many different entities. These findings identify GLUT1 as a promising prognostic cancer marker, which may be clinically useful in many cancer types.

## Supplementary Information

Supplementary Material 1: Supplementary Figure 1. Assay validation by comparison of two antibodies. The panels show immunostaining results obtained by two independent GLUT1 antibodies. Using MSVA-401R, a staining was seen in a subset of epithelial cells of the rectum (A), germinal center cells of the tonsil (B), a subset of epithelial cells of the seminal vesicle (C), some epithelial cells of the fallopian tube (D), decidua cells of the placenta (E), a subset of collecting ducts of the kidney (F), trophoblast cells of the mature placenta (G), and the basal and suprabasal cell layers of the urothelium (H). Using clone 355A-15, a comparable but weaker staining was seen in the rectum (a), the tonsil (b), the seminal vesicle (c), the fallopian tube (d), the placenta (e), the kidney (f), the placenta (g), and the urothelium (h). The images A-H and a-h are from consecutive tissue sections.

Supplementary Material 2. Supplementary Table 1. GLUT1 immunostaining in previous studies.

Supplementary Material 3. Supplementary Table 2. Gender and age of the patients, where available. N/A = Not assessed.

Supplementary Material 4. Supplementary Table 3. Multivariate analysis of GLUT1 immunostaining versus tumor stage andgrade in clear cell renal cell carcinomas.

## Data Availability

All data generated or analyzed during this study are included in this published article.

## Abbreviations

GLUT: Glucose transporter family
IHC: Immunohistochemistry
IWGAV: International Working Group of Antibody Validation
MMR: Mismatch repair
TEC: Tris-EDTA-Citrat
TMA: Tissue microarray
