# Supplementary figures and images for: Glucose-transporter 1 (GLUT1) as a prognostic biomarker: evidence from 14,966 human tumors across 134 cancer types
Source: BMC Cancer. 2026 Jan 10;26:127. doi: 10.1186/s12885-025-15527-5 (PMC12836948; doi:10.1186/s12885-025-15527-5)

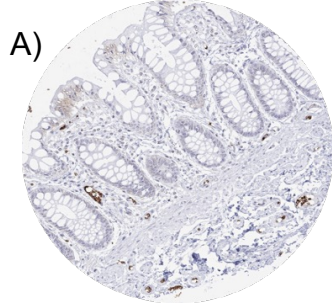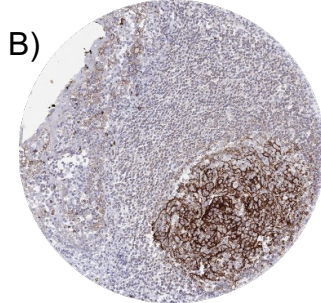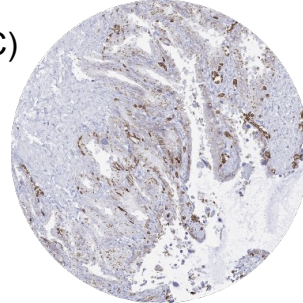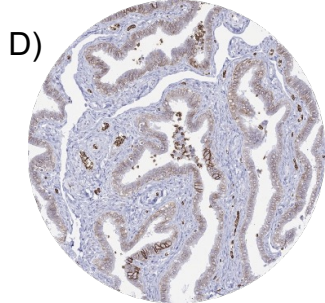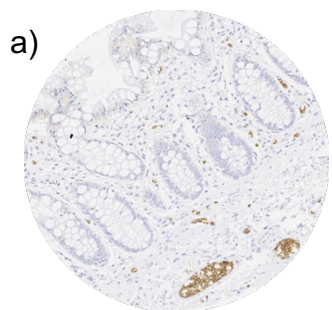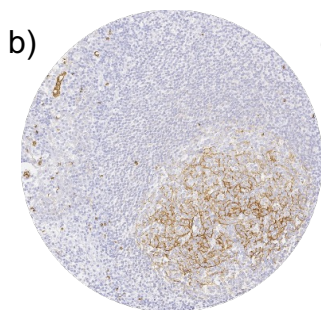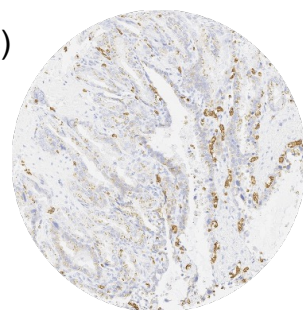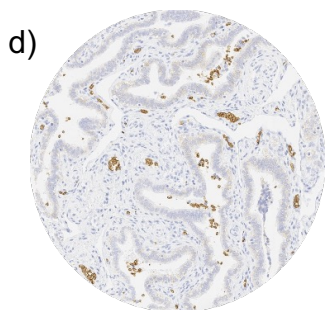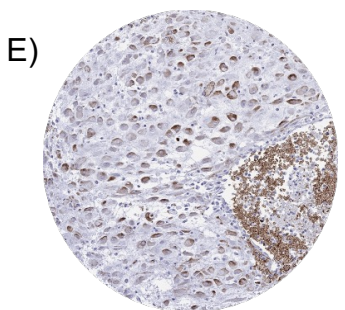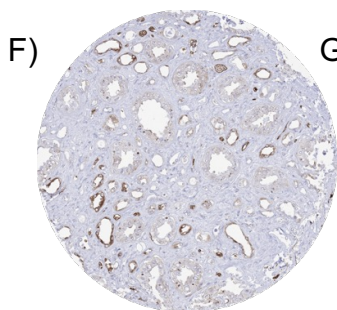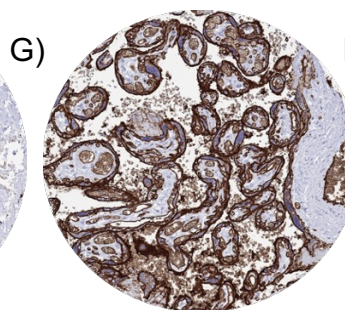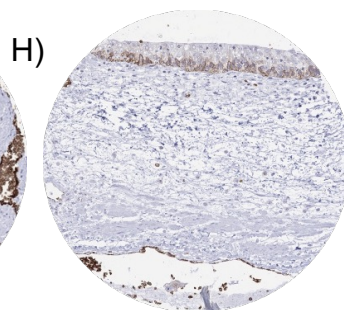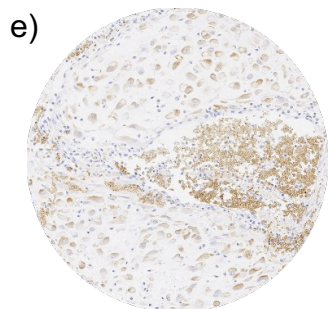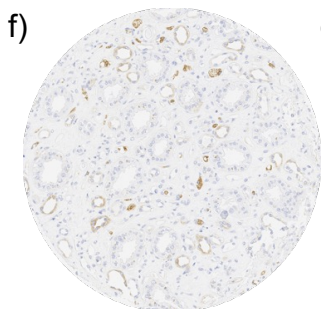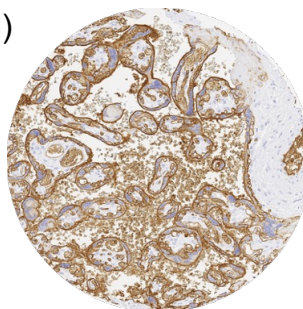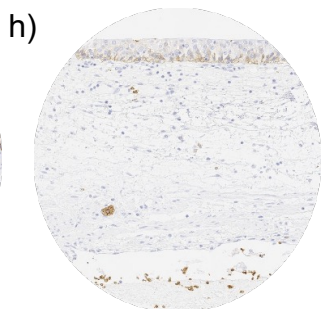

Supplement: Supplementary file 1 — Supplementary Material 1: Supplementary Figure 1. Assay validation by comparison of two antibodies. The panels show immunostaining results obtained by two independent GLUT1 antibodies. Using MSVA-401R, a staining was seen in a subset of epithelial cells of the rectum (A), germinal center cells of the tonsil (B), a subset of epithelial cells of the seminal vesicle (C), some epithelial cells of the fallopian tube (D), decidua cells of the placenta (E), a subset of collecting ducts of the kidney (F), trophoblast cells of the mature placenta (G), and the basal and suprabasal cell layers of the urothelium (H). Using clone 355A-15, a comparable but weaker staining was seen in the rectum (a), the tonsil (b), the seminal vesicle (c), the fallopian tube (d), the placenta (e), the kidney (f), the placenta (g), and the urothelium (h). The images A-H and a-h are from consecutive tissue sections. [file 12885_2025_15527_MOESM1_ESM.pdf]
