# Supplementary material for: Glucose-transporter 1 (GLUT1) as a prognostic biomarker: evidence from 14,966 human tumors across 134 cancer types
Source: BMC Cancer. 2026 Jan 10;26:127. doi: 10.1186/s12885-025-15527-5 (PMC12836948; doi:10.1186/s12885-025-15527-5)
Supplement: Supplementary file 2 — Supplementary Material 2. Supplementary Table 1. GLUT1 immunostaining in previous studies. [file 12885_2025_15527_MOESM2_ESM.pdf]

Supplementary table 1. GLUT1 immunostaining in previous studies

author	PMID	tumor entity	analyzed tumors (n)	GLUT1 positiv (%)
Younes M, et al.	9149888	Adenocarcinoma of the esophagus	13	69.23%
Higashi T, et al.	9293783	Ductal adenocarcinoma of the pancreas	21	85.71%
Higashi T, et al.	9293783	Pancreatic/Ampullary adenocarcinoma	1	100.00%
Higashi T, et al.	9776278	Ductal adenocarcinoma of the pancreas	24	87.50%
Higashi T, et al.	9776278	Pancreatic/Ampullary adenocarcinoma	3	100.00%
Rao UN, et al.	10574596	Leiomyosarcoma	16	0.00%
Rao UN, et al.	10574596	Leiomyosarcoma	14	0.00%
Chang S, et al.	10699635	Urothelial carcinoma, pT2-4 G3	31	67.74%
Loda M, et al.	10736070	Rhabdoid tumor	2	100.00%
Kawamura T, et al.	11505409	Gastric adenocarcinoma, intestinal type	16	6.25%
Kawamura T, et al.	11505409	Gastric adenocarcinoma, intestinal type	80	43.75%
Kawamura T, et al.	11505409	Gastric adenocarcinoma, diffuse type	176	28.41%
Kawamura T, et al.	11505409	Gastric adenocarcinoma, intestinal type	196	48.47%
Kawamura T, et al.	11505409	Gastric adenocarcinoma, diffuse type	49	2.04%
Cantuaria G, et al.	11571727	Serous carcinoma of the ovary	71	88.73%
Cantuaria G, et al.	11571727	Mucinous carcinoma of the ovary	3	66.66%
Cantuaria G, et al.	11571727	Endometrioid carcinoma of the ovary	16	75.00%
Lambert DW, et al.	11953883	Adenocarcinoma of the colon	24	83.33%
Kim YW, et al.	12143238	Gallbladder adenocarcinoma	71	52.11%
Mineta H, et al.	12552944	Squamous cell carcinoma of the pharynx	99	46.46%
Koga H, et al.	12932118	Diffuse large B cell lymphoma (DLBCL)	1	100.00%
Cooper R, et al.	12942120	Adenocarcinoma of the colon	43	69.77%
Kurokawa T, et al.	15027127	Endometrioid carcinoma of the ovary	2	100.00%
Kurokawa T, et al.	15027127	Mucinous carcinoma of the ovary	5	60.00%
Kurokawa T, et al.	15027127	Serous carcinoma of the ovary	9	88.89%
Weiner MF, et al.	15468132	ral squamous cell carcinoma (floor of the mout	5	100.00%
Weiner MF, et al.	15468132	Squamous cell carcinoma of the larynx	3	100.00%
Weiner MF, et al.	15468132	Squamous cell carcinoma of the pharynx	2	100.00%
Jonathan RA, et al.	16730088	ral squamous cell carcinoma (floor of the mout	5	0.00%
Jonathan RA, et al.	16730088	Squamous cell carcinoma of the pharynx	11	9.09%
Kim YW, et al.	17029809	Anaplastic thyroid carcinoma	7	85.71%
Kato Y, et al.	17192790	Mesothelioma, biphasic	1	100.00%
Yamada A, et al.	17294670	Gastric adenocarcinoma, intestinal type	13	69.23%
Yamada A, et al.	17294670	Gastric adenocarcinoma, diffuse type	3	0.00%
Yamada A, et al.	17294670	Gastric adenocarcinoma, intestinal type	2	100.00%
Yamada A, et al.	17294670	Gastric adenocarcinoma, diffuse type	9	22.22%
Yamada A, et al.	17294670	Gastric adenocarcinoma, intestinal type	4	100.00%
Cleven AH, et al.	17452775	Adenocarcinoma of the colon	133	84.96%
Riedel CC, et al.	17475966	Adenocarcinoma of the colon	90	65.56%
Lidgren A, et al.	17922867	Papillary renal cell carcinoma	23	100.00%
Yasuda M, et al.	18097583	Clear cell carcinoma of the ovary	18	100.00%
Yasuda M, et al.	18097583	Endometrioid carcinoma of the ovary	16	100.00%
Yasuda M, et al.	18097583	Mucinous carcinoma of the ovary	19	100.00%
Yasuda M, et al.	18097583	Serous carcinoma of the ovary	21	100.00%
Ozbudak IH, et al.	18191183	Endometrioid endometrial carcinoma	49	73.47%
Ozbudak IH, et al.	18191183	Endometrioid endometrial carcinoma	20	100.00%
Ahrens WA, et al.	18620729	Chondrosarcoma	5	0.00%
Ahrens WA, et al.	18620729	Dermatofibrosarcoma protuberans	10	0.00%
Ahrens WA, et al.	18620729	alignant peripheral nerve sheath tumor (MPNS	3	33.33%
Ahrens WA, et al.	18620729	Solitary fibrous tumor	11	9.09%
Ahrens WA, et al.	18620729	Synovial sarcoma	10	30.00%
Schrijvers ML, et al.	18722267	Squamous cell carcinoma of the larynx	91	100.00%
Salla JT, et al.	18826382	alignant peripheral nerve sheath tumor (MPNS	4	25.00%
Heinricht S, et al.	19092346	Pancreatic/Ampullary adenocarcinoma	22	100.00%
Ozbudak IH, et al.	19234439	Lung, neuroendocrine tumor (NET)	29	21.00%
Ozbudak IH, et al.	19234439	Lung, neuroendocrine tumor (NET)	23	74.00%
Ozbudak IH, et al.	19234439	Lung, neuroendocrine tumor (NET)	46	7.00%
Jung YH, et al.	19235753	Follicular thyroid carcinoma	7	14.29%
Jung YH, et al.	19235753	Papillary thyroid carcinoma	14	28.57%
Jung YH, et al.	19235753	Anaplastic thyroid carcinoma	3	100.00%
Khandani AH, et al.	19536037	Hodgkin's lymphoma	1	100.00%
Khandani AH, et al.	19536037	Hodgkin's lymphoma	3	100.00%
Khandani AH, et al.	19536037	Follicular lymphoma	3	100.00%
Khandani AH, et al.	19536037	Follicular lymphoma	4	100.00%
Kojika M, et al.	19648882	Thymoma	84	100.00%
Airley R, et al.	20395120	Adenocarcinoma of the lung	2	0.00%
Airley R, et al.	20395120	Squamous cell carcinoma of the lung	2	100.00%
Airley R, et al.	20395120	Clear cell carcinoma of the ovary	5	20.00%
Airley R, et al.	20395120	Invasive breast carcinoma of no special type	32	18.75%
Airley R, et al.	20395120	Endometrioid carcinoma of the ovary	5	40.00%
Airley R, et al.	20395120	Adenocarcinoma of the colon	4	0.00%
Airley R, et al.	20395120	Serous carcinoma of the ovary	12	58.33%
Airley R, et al.	20395120	Serous carcinoma of the ovary	26	42.31%
Yiong Y, et al.	20527231	Endometrioid endometrial carcinoma	24	91.67%
Krockenberger M, et al	20592357	Adenocarcinoma of the cervix	41	95.12%
Mayer A, et al.	21109935	Squamous cell carcinoma of the cervix	51	0.00%
Semaan A, et al.	21167567	Serous carcinoma of the ovary	204	100.00%
Korkeila E, et al.	21273616	Adenocarcinoma of the colon	175	54.29%
Rohan SM, et al.	21602815	Clear cell (tubulo) papillary renal cell carcinoma	9	100.00%
Rohan SM, et al.	21602815	Papillary renal cell carcinoma	13	84.62%
Carvalho KC, et al.	21808860	Squamous cell carcinoma of the cervix	164	42.07%
Han MW, et al.	22052623	ral squamous cell carcinoma (floor of the mout	33	69.70%
Kawada K, et al.	22282467	Adenocarcinoma of the colon	63	80.95%
Kaira K, et al.	22329847	Lung, neuroendocrine tumor (NET)	34	79.41%
Kaira K, et al.	22329847	Thymoma	49	32.65%
Matter A, et al.	22494537	Schwannoma	1	0.00%
Chlumská A, et al.	22716059	Ganglioneuroma	1	100.00%
Lagana SM, et al.	22742553	Mesothelioma, biphasic	35	62.86%
Lagana SM, et al.	22742553	Mesothelioma, epithelioid	100	51.00%
Lagana SM, et al.	22742553	Mesothelioma, biphasic	9	77.78%
Lagana SM, et al.	22742553	Mesothelioma, epithelioid	21	38.10%
Mitchell A, et al.	22762889	alignant peripheral nerve sheath tumor (MPNS	1	100.00%
Zámečník M, et al.	23121031	Ganglioneuroma	8	100.00%
Hayashi A, et al.	23801129	Thymoma	17	47.06%
Pontes HA, et a.	23969921	T-cell non-Hodgkin's lymphoma	1	0.00%
Adam P, et al.	24496993	Adenocarcinoma of the colon	1	100.00%
Kitasato Y, et al.	25121413	Ductal adenocarcinoma of the pancreas	41	75.61%
Pinheiro C, et al.	25296855	Squamous cell carcinoma of the cervix	29	65.52%
Yu YH, et al.	25747176	ral squamous cell carcinoma (floor of the mout	32	100.00%
Stagner AM, et al.	25794028	Neurofibroma	5	100.00%
Weijer R, et al.	26657503	Cholangiocarcinoma	2	100.00%
Canpolat T, et al.	27562387	Endometrioid endometrial carcinoma	100	95.00%
Wang T, et al.	29463225	Adenocarcinoma of the colon	81	100.00%
Vermeulen MA, et al.	31653758	Invasive breast carcinoma of no special type	58	75.86%
Vermeulen MA, et al.	31653758	Invasive breast carcinoma of no special type	18	61.11%
Vermeulen MA, et al.	31653758	Invasive breast carcinoma of no special type	58	62.07%
Shen N, et al.	32533646	Diffuse large B cell lymphoma (DLBCL)	39	64.10%
Fujishima F, et al.	33851620	Mesothelioma, epithelioid	6	0.00%
Dalle Fratte C, et al.	34108073	Adenocarcinoma of the colon	95	0.00%
Berklite L, et al.	37277077	Angiosarcoma	3	100.00%
Nagase Y, et al.	7861542	Clear cell renal cell carcinoma	52	84.62%
Ito T, et al.	9619596	Adenocarcinoma of the lung	24	58.33%
Cantuaria G, et al.	11006027	Serous carcinoma of the ovary	64	100.00%
Cantuaria G, et al.	11006027	Mucinous carcinoma of the ovary	16	100.00%
Baer S, et al.	11889403	Squamous cell carcinoma of the larynx	48	100.00%
Miyakita H, et al.	11972644	Clear cell renal cell carcinoma	15	93.33%
Minami K, et al.	12367793	Adenocarcinoma of the lung	47	40.43%
Kato H, et al.	12529975	Squamous cell carcinoma of the esophagus	95	51.58%
Tomes L, et al.	14531498	Invasive breast carcinoma of no special type	48	90.00%
Tian M, et al	14551748	ral squamous cell carcinoma (floor of the mout	19	94.74%
Oliver RJ, et al.	14962715	ral squamous cell carcinoma (floor of the mout	54	90.74%
Tohma T, et al.	16045581	Squamous cell carcinoma of the esophagus	63	100.00%
Yasuda M, et al.	16273245	Papillary thyroid carcinoma	129	70.54%
Yasuda M, et al.	16273245	Follicular thyroid carcinoma	60	5.00%
Jonathan RA, et al.	16730088	Squamous cell carcinoma of the larynx	42	19.05%
Koukourakis MI, et al.	16827797	Adenocarcinoma of the colon	79	100.00%
Kim YW, et al.	17029809	Papillary thyroid carcinoma	20	0.00%
Kim YW, et al.	17029809	Follicular thyroid carcinoma	40	0.00%
Kim YW, et al.	17029809	Medullary thyroid carcinoma	10	0.00%
de Geus-Oei LF, et al.	17046099	Squamous cell carcinoma of the lung	8	100.00%
de Geus-Oei LF, et al.	17046099	Adenocarcinoma of the lung	8	100.00%
Kato Y, et al.	17192790	Mesothelioma, epithelioid	36	100.00%
Kato Y, et al.	17192790	Mesothelioma, biphasic	11	63.64%
Kato Y, et al.	17192790	Adenocarcinoma of the lung	30	93.33%
Kato Y, et al.	17192790	Squamous cell carcinoma of the lung	28	100.00%
kunekl m, et al.	17207657	ral squamous cell carcinoma (floor of the mout	40	100.00%
Sun HC, et al.	17487356	Ductal adenocarcinoma of the pancreas	58	67.20%
Ozbudak IH, et al.	18191183	Endometrial carcinoma, high grade, G3	31	100.00%
Paudyal B, et al.	18250992	Hepatocellular carcinoma	1	0.00%
Ahrens WA, et al.	18620729	Angiomyolipoma	3	0.00%
Ahrens WA, et al.	18620729	Angiosarcoma	2	0.00%
Ahrens WA, et al.	18620729	Ewing sarcoma	11	27.27%
Ahrens WA, et al.	18620729	Granular cell tumor	1	100.00%
Ahrens WA, et al.	18620729	Gastrointestinal stromal tumor (GIST)	14	14.29%
Ahrens WA, et al.	18620729	Leiomyosarcoma	10	40.00%
Ahrens WA, et al.	18620729	Liposarcoma	10	0.00%
Ahrens WA, et al.	18620729	Leiomyoma	1	0.00%
Ahrens WA, et al.	18620729	Myofibrosarcoma	1	0.00%
Ahrens WA, et al.	18620729	Neurofibroma	11	36.36%
Ahrens WA, et al.	18620729	Rhabdomyosarcoma	4	75.00%
Ahrens WA, et al.	18620729	Schwannoma	18	22.22%
Ahrens WA, et al.	18620729	Osteosarcoma	2	0.00%
Salla JT, et al.	18826382	Neurofibroma	11	81.82%
Eckert AW, et al.	19020718	ral squamous cell carcinoma (floor of the mout	42	23.81%
Khandani AH, et al.	19536037	Diffuse large B cell lymphoma (DLBCL)	9	100.00%
Khandani AH, et al.	19536037	Burkitt Lymphoma	3	100.00%
Ogane N, et al.	20123453	Squamous cell carcinoma of the esophagus	96	100.00%
Miyawaki A, et al	20372831	ral squamous cell carcinoma (floor of the mout	37	81.08%
Airley R, et al.	20395120	Squamous cell carcinoma of the lung	25	88.00%
Airley R, et al.	20395120	Adenocarcinoma of the lung	17	64.71%
Airley R, et al.	20395120	Adenocarcinoma of the colon	41	53.66%
Airley R, et al.	20395120	Lobular carcinoma of the breast	7	0.00%
Airley R, et al.	20395120	Mucinous carcinoma of the ovary	5	0.00%
Airley R, et al.	20395120	Clear cell carcinoma of the ovary	6	33.33%
Legan M, et al.	20512538	Gallbladder adenocarcinoma	56	48.21%
Seeber LM, et al.	20565904	Endometrioid endometrial carcinoma	93	81.72%
Singer K, et al.	20607826	Clear cell renal cell carcinoma	79	27.00%
Singer K, et al.	20607826	Papillary renal cell carcinoma	66	24.24%
Singer K, et al.	20607826	Chromophobe renal cell carcinoma	73	28.77%
Singer K, et al.	20607826	Oncocytoma of the kidney	30	0.00%
Eckert AW, et al.	21438910	ral squamous cell carcinoma (floor of the mout	79	59.49%
Rohan SM, et al.	21602815	Clear cell renal cell carcinoma	11	100.00%
Ohno T, et al.	21773906	Schwannoma	2	0.00%
Moon SH, et al.	22888973	Papillary thyroid carcinoma	40	0.00%
Li YZ, et al.	23098453	Squamous cell carcinoma of the vulva	25	100.00%
Kita Y, et al.	23238695	Squamous cell carcinoma of the esophagus	80	30.00%
Souto GR, et al.	23336292	Granular cell tumor	6	0.00%
Requena L, et al.	23598966	alignant peripheral nerve sheath tumor (MPNS	3	0.00%
Requena L, et al.	23598966	Basal cell carcinoma of the skin	1	0.00%
Hayashi A, et al.	23801129	Squamous cell carcinoma of the lung	12	100.00%
Shimada Y, et al.	24096997	Schwannoma	1	100.00%
Huang XQ, et al.	24817962	Squamous cell carcinoma of the cervix	132	100.00%
Alves VA, et al.	25206282	Hepatocellular carcinoma	76	9.21%
Pinheiro C, et al.	25296855	Squamous cell carcinoma of the cervix	37	75.68%
Angadi VC, et al.	26062860	ral squamous cell carcinoma (floor of the mout	30	100.00%
Choi WH, et al.	26410497	Adenocarcinoma of the lung	26	88.46%
Choi WH, et al.	26410497	Squamous cell carcinoma of the lung	35	100.00%
Cho MH, et al.	26509967	Gastrointestinal stromal tumor (GIST)	40	90.00%
Pinheiro C, et al.	26525902	Adenocarcinoma of the cervix	197	44.16%
Pereira KM, et al.	26838200	ral squamous cell carcinoma (floor of the mout	15	100.00%
Khanom R, et al.	27512993	ral squamous cell carcinoma (floor of the mout	50	84.00%
Higashi K, et al.	27663442	Adenocarcinoma of the lung	44	43.18%
Lodewijk L, et al.	28404916	Medullary thyroid carcinoma	111	5.41%
Li YC, et al.	29398870	Hepatocellular carcinoma	55	100.00%
Kurahara H, et al.	29404819	Ductal adenocarcinoma of the pancreas	100	100.00%
Lastraoli E, et al.	31679954	Clear cell renal cell carcinoma	30	23.33%
Achalandabaso Boira M	32967636	Ductal adenocarcinoma of the pancreas	39	100.00%
Gill RM, et al.	27090685	Angiosarcoma	6	33.00%
Michal M, et al.	28325366	Synovial sarcoma	4	100.00%
Brooks S A, et al.	26787754	Clear cell renal cell carcinoma	10	100.00%
Daskalow K, et al.	19350262	Hepatocellular carcinoma	58	0.00%
Hirose T, et al.	12692193	alignant peripheral nerve sheath tumor (MPNS	11	55.00%
Koukourakis M I, et al.	16423989	Adenocarcinoma of the colon	70	80.00%
Shim H K, et al.	19217531	Diffuse large B cell lymphoma (DLBCL)	11	0.00%
Kwon O J, et al.	24677746	Squamous cell carcinoma of the larynx	42	67.00%
Chen R, et al.	30723389	Clear cell renal cell carcinoma	54	20.00%
Izquierdo F, et al.	22668577	Granular cell tumor	293	32.00%
Schmitt A M, et al.	19690016	Pancreas, neuroendocrine tumor (NET)	152	30.00%
North P E, et al.	10665907	Angiosarcoma	12	25.00%
Krawczyk M A, et al.	30995126	Rhabdomyosarcoma	53	38.00%
Greijer A E, et al.	18351386	Adenocarcinoma of the colon	23	100.00%
Blank A, et al.	20702724	Pheochromocytoma	112	7.10%
Kaida H., et al.	20548142	Gastrointestinal stromal tumor (GIST)	1	100.00%
Husain A N., et al.	24440278	Mesothelioma, epithelioid	41	50.00%
Husain A N., et al.	24440278	Mesothelioma, biphasic	3	100.00%
Amano S., et al.	38082268	Pancreas, neuroendocrine tumor (NET)	47	51.00%
Yamashita K., et al.	34635937	alignant peripheral nerve sheath tumor (MPNS	3	100.00%
Fan J., et al.	28781680	Osteosarcoma	51	74.00%
Chang M S., et al.	24020724	Squamous cell carcinoma of the cervix	4	100.00%
Charles N C., et al.	37338324	Neurofibroma	1	100.00%
Legan M., et al.	19606394	Gallbladder adenocarcinoma	57	53.00%
Hyseni A., et al.	21732131	Invasive breast carcinoma of no special type	92	26.00%
Suteau V., et al.	35053598	Invasive breast carcinoma of no special type	144	60.00%
Friedrich R E., et al.	22593502	Neurofibroma	15	100.00%
Horree N., et al.	17452774	Endometrioid endometrial carcinoma	39	95.00%
Grabellus F., et al.	22376167	Papillary thyroid carcinoma	57	68.00%
Fujinaga A., et al.	37193984	Cholangiocarcinoma	39	44.00%
Schraml P., et al	18660794	Clear cell renal cell carcinoma	18	78.00%
Schraml P., et al	18660794	Papillary renal cell carcinoma	9	0.00%
Hirose Y., et al.	23701133	Diffuse large B cell lymphoma (DLBCL)	52	76.00%
Zhao X., et al.	34502178	Squamous cell carcinoma of the esophagus	30	83.00%
Hussein Y R., et al.	22190995	Invasive breast carcinoma of no special type	284	37.00%
Hussein Y R., et al.	22190995	Lobular carcinoma of the breast	26	4.00%
Kushitani K., et al.	26735863	Mesothelioma, epithelioid	66	91.00%
Gomez-Brouchet A., et	35158767	Osteosarcoma	98	49.00%
Kim K., et al.	23323103	Serous carcinoma of the ovary	63	51.00%
Kim K., et al.	23323103	Mucinous carcinoma of the ovary	24	46.00%
Kim K., et al.	23323103	Clear cell carcinoma of the ovary	25	52.00%
Kim K., et al.	23323103	Endometrioid carcinoma of the ovary	11	36.00%
Higashi K., et al.	24578007	Adenocarcinoma of the lung	23	96.00%
Higashi K., et al.	24578007	Squamous cell carcinoma of the lung	4	100.00%
Boström P J., et al.	27376131	Urothelial carcinoma, pT2-4 G3	279	79.00%
Chang S., et al.	25376377	Mesothelioma, epithelioid	59	15.00%
Chang S., et al.	25376377	Mesothelioma, biphasic	17	35.00%
Bonatelli M., et al.	31316469	Seminoma	63	93.00%
Ito R., et al.	36844619	Adenocarcinoma of the lung	299	37%
Ito R., et al.	36844619	Squamous cell carcinoma of the lung	120	55%
Ma X., et al.	26101475	Squamous cell carcinoma of the cervix	60	70.00%
Lastraioli E., et al.	22496927	Adenocarcinoma of the colon	135	35.00%
Ibrahiem A T., et al.	35308569	Papillary thyroid carcinoma	51	85.00%
Nakazato K., et al.	31423200	ral squamous cell carcinoma (floor of the mout	220	100.00%
Blayney J K., et al.	29719622	Adenocarcinoma of the esophagus	141	81.00%
Wang L., et al.	37892091	Ductal adenocarcinoma of the pancreas	102	100.00%
Jang S M., et al.	22807934	Invasive breast carcinoma of no special type	106	38.00%
Seleit I., et al.	28764171	Basal cell carcinoma of the skin	20	100.00%
Seleit I., et al.	28764171	Squamous cell carcinoma of the skin	20	100.00%
Iwasaki K., et al.	26622782	Squamous cell carcinoma of the cervix	34	82.35%
Iwasaki K., et al.	26622782	Adenocarcinoma of the cervix	20	60.00%
Lastraioli E, et al.	34359733	Adenocarcinoma of the colon	86	37.21%
Murtori L, et al.	27789963	Adenocarcinoma of the colon	162	34.57%
Choi YS, et al.	19746220	ral squamous cell carcinoma (floor of the mout	60	100.00%
Aboushousha T, et al.	29373917	Hepatocellular carcinoma	33	87.88%
Lewitowicz P, et al.	27795705	Gastrointestinal stromal tumor (GIST)	54	0.00%
Kim S, et al.	23843845	Mucinous carcinoma of the breast	59	27.12%
Lee JW, et al.	26085858	Papillary thyroid carcinoma	46	100.00%
Kwee SA, et al.	27069767	Hepatocellular carcinoma	5	100.00%
Iorio J, et al.	32105990	Adenocarcinoma of the colon	80	77.50%
Guo Y, et al.	36561502	Gastric adenocarcinoma, intestinal type	13	100.00%
Kumari A, et al.	36989865	ral squamous cell carcinoma (floor of the mout	120	100.00%
Thamrongwaranggoon	33402473	Cholangiocarcinoma	82	100.00%
Thamrongwaranggoon	33402473	Cholangiocarcinoma	27	66.67%
Thamrongwaranggoon	33402473	Cholangiocarcinoma	55	89.09%
Kim EK, et al.	34518477	Adrenal cortical adenoma	115	28.70%
Kim EK, et al.	34518477	Adrenal cortical carcinoma	17	52.94%
Ahopelto K, et al.	32434192	Gastric adenocarcinoma, diffuse type	161	36.65%
Ahopelto K, et al.	32434192	Gastric adenocarcinoma, intestinal type	111	77.48%
Koh YW, et al.	28212997	Adenocarcinoma of the lung	76	39.47%
Koh YW, et al.	28212997	Adenocarcinoma of the lung	9	11.11%
Koh YW, et al.	28212997	Adenocarcinoma of the lung	26	42.31%
Koh YW, et al.	28212997	Adenocarcinoma of the lung	6	83.33%
Koh YW, et al.	28212997	Adenocarcinoma of the lung	32	81.25%
Koh YW, et al.	28212997	Adenocarcinoma of the lung	8	62.50%
Koh YW, et al.	28212997	Squamous cell carcinoma of the lung	93	99.00%
Liu Y, et al.	32510678	Mesothelioma, epithelioid	30	80.00%
Sun et al.	28036362	Hepatocellular carcinoma	96	100.00%
Chen HL,et al.	30306706	Hepatocellular carcinoma	213	100.00%
Zuo J, et al.	26749588	Squamous cell carcinoma of the larynx	57	21.05%
Zhou YX, et al.	29629851	Squamous cell carcinoma of the esophagus	117	65.81%
Shim BY, et al.	22847606	Adenocarcinoma of the colon	104	24.04%
Sawayama H, et al.	30861255	Squamous cell carcinoma of the esophagus	97	50.52%
Sawayama H, et al.	30861255	Squamous cell carcinoma of the esophagus	61	52.46%
Du MJ, et al.	27688088	Thymoma	21	38.10%
Du J, et al.	34229540	Ductal adenocarcinoma of the pancreas	88	100.00%
Du J, et al.	34229540	Ductal adenocarcinoma of the pancreas	76	100.00%
Schlößer HA, et al.	26643879	Gastric adenocarcinoma, diffuse type	90	8.89%
Schlößer HA, et al.	26643879	Gastric adenocarcinoma, intestinal type	60	86.67%
Yin C, et al.	32201432	Gastric adenocarcinoma, diffuse type	102	42.16%
Yin C, et al.	32201432	Gastric adenocarcinoma, intestinal type	132	69.70%
Maki Y, et al.	23076555	Adenocarcinoma of the lung	105	26.67%
Kim SJ, et al.	18324916	Pancreatic/Ampullary adenocarcinoma	36	58.33%
Haber RS, et al.	9226204	Papillary thyroid carcinoma	17	52.94%
Haber RS, et al.	9226204	Follicular thyroid carcinoma	6	33.33%
Haber RS, et al.	9226204	Anaplastic thyroid carcinoma	2	100.00%
Haber RS, et al.	9226204	Medullary thyroid carcinoma	2	0.00%
Kaira K, et al.	27268922	ral squamous cell carcinoma (floor of the mout	85	100.00%
Kasahara N, et al.	30089592	Squamous cell carcinoma of the lung	167	100.00%
Na S , et al.	29080392	ral squamous cell carcinoma (floor of the mout	45	100.00%
Berlth F, et al.	24982335	Gastric adenocarcinoma, intestinal type	218	99.08%
Ayala FR, et al.	20428049	ral squamous cell carcinoma (floor of the mout	142	95.07%
Wincewicz A, et al.	17387384	Adenocarcinoma of the colon	107	70.09%
Wincewicz A, et al.	17387384	Adenocarcinoma of the colon	64	73.44%
Shan X, et al.	23321829	Lobular carcinoma of the breast	2	0.00%
de Carvalho PA, et al.	34668521	Clear cell renal cell carcinoma	204	77.45%
Melstrom LG, et al.	21227456	Ductal adenocarcinoma of the pancreas	15	100.00%
Kobayashi M, et al.	22475893	Squamous cell carcinoma of the esophagus	57	59.65%
Grabellus F, et al.	22228332	Adenoma of the thyroid gland	20	90.00%
Grabellus F, et al.	22228332	Follicular thyroid carcinoma	17	58.82%
Grabellus F, et al.	22228332	Papillary thyroid carcinoma	20	80.00%
Grabellus F, et al.	22228332	Anaplastic thyroid carcinoma	18	100.00%
Osugi J, et al.	25837797	Squamous cell carcinoma of the lung	56	80.36%
UsudaK, et al.	20703861	Adenocarcinoma of the lung	17	64.71%
UsudaK, et al.	20703861	Squamous cell carcinoma of the lung	12	100.00%
Cai Y, et al.	25056472	Serous carcinoma of the ovary	40	67.50%
Cai Y, et al.	25056472	Serous carcinoma of the ovary	40	97.50%
Cai Y, et al.	25056472	Serous carcinoma of the ovary	80	100.00%
Kang DY, et al.	19154259	Squamous cell carcinoma of the lung	61	86.89%
Kang DY, et al.	19154259	Adenocarcinoma of the lung	64	18.75%
Brown RS, et al.	10210213	Adenocarcinoma of the lung	6	100.00%
Khabaz MN, et al.	31686415	Endometrial clear cell carcinoma	1	100.00%
Khabaz MN, et al.	31686415	Endometrioid endometrial carcinoma	59	88.14%
Khabaz MN, et al.	31686415	Endometrial serous carcinoma	9	100.00%
Laudański P, et al.	15254667	Lobular carcinoma of the breast	12	41.67%
Sasaki H, et al.	22200795	Adenocarcinoma of the lung	189	35.98%
Sukeda A, et al.	31404019	Ductal adenocarcinoma of the pancreas	240	15.00%
Brown RS, et al.	12031879	Lobular carcinoma of the breast	7	42.86%
Pinheiro C, et al.	27105345	Malignant melanoma lymph node metastasis	111	21.62%
Pinheiro C, et al.	27105345	Malignant melanoma lymph node metastasis	54	0.00%
Andersen S, et al.	21075472	Squamous cell carcinoma of the lung	191	97.38%
Andersen S, et al.	21075472	Adenocarcinoma of the lung	113	94.69%
Yoon M, et al.	26513490	Papillary thyroid carcinoma	52	82.69%
Önder S, et al.	31165505	Mesothelioma, epithelioid	68	67.65%
Önder S, et al.	31165505	Mesothelioma, biphasic	12	66.67%
Sawayama H, et al.	24242681	Squamous cell carcinoma of the esophagus	145	28.28%
Benzerdjeb N, et al.	27995675	Lung, neuroendocrine tumor (NET)	18	100.00%
Afonso J, et al.	36724756	Adenocarcinoma of the esophagus	44	29.55%
Afonso J, et al.	36724756	Squamous cell carcinoma of the esophagus	49	81.63%
Hommell-Fontaine J, e	23800898	Mesothelioma, epithelioid	28	57.14%
Mapelli P, et al.	29119942	Adenocarcinoma of the lung	6	50.00%
Mapelli P, et al.	29119942	Squamous cell carcinoma of the lung	1	100.00%
Hur H, et al.	23135628	Gastric adenocarcinoma, diffuse type	62	11.29%
Hur H, et al.	23135628	Gastric adenocarcinoma, intestinal type	58	36.21%
Hur H, et al.	23135628	Gastric adenocarcinoma, mixed type	21	23.81%
Hong R, et al.	22253523	Adenocarcinoma of the colon	44	61.36%
Roh MS, et al.	15362741	Hepatocellular carcinoma	22	4.55%
Roh MS, et al.	15362741	Cholangiocarcinoma	16	81.25%
Vermeulen JF, et al.	22695343	Lobular carcinoma of the breast	126	3.97%
Abdou AG, et al.	26092229	Squamous cell carcinoma of the skin	16	100.00%
Abdou AG, et al.	26092229	Basal cell carcinoma of the skin	16	62.50%
Hiyoshi Y, et al.	19262068	Squamous cell carcinoma of the esophagus	60	100.00%
Tsukioka M, et al.	25421331	Serous carcinoma of the ovary	58	100.00%
Tsukioka M, et al.	25421331	Mucinous carcinoma of the ovary	20	100.00%
Tsukioka M, et al.	25421331	Endometrioid carcinoma of the ovary	24	100.00%
Tsukioka M, et al.	25421331	Clear cell carcinoma of the ovary	49	95.92%
Jung JH, et al.	23935399	Gastric adenocarcinoma, diffuse type	63	30.16%
Jung JH, et al.	23935399	Gastric adenocarcinoma, intestinal type	130	49.23%
Wang BY, et al.	10870060	Endometrioid endometrial carcinoma	41	100.00%
Kaira K, et al.	22055682	Schwannoma	6	50.00%
Kaira K, et al.	22055682	Teratoma	3	66.67%
Kaira K, et al.	22055682	Seminoma	1	0.00%
Kaira K, et al.	22055682	Ganglioneuroma	1	0.00%
Kaira K, et al.	22055682	Hodgkin's lymphoma	1	100.00%
Kim WS, et al.	10983690	Gastric adenocarcinoma, diffuse type	38	5.26%
Kim WS, et al.	10983690	Gastric adenocarcinoma, intestinal type	27	33.33%
Pizzi S, et al.	19085834	Ductal adenocarcinoma of the pancreas	60	100.00%
Alakus H, et al.	20220543	Gastric adenocarcinoma, diffuse type	12	91.67%
Alakus H, et al.	20220543	Gastric adenocarcinoma, intestinal type	20	40.00%
Alakus H, et al.	20220543	Gastric adenocarcinoma, mixed type	3	66.67%
Kubo Y, et al.	24824030	Cholangiocarcinoma	149	46.31%
Kaida H, et al.	21606885	Papillary thyroid carcinoma	54	44.44%
Grimm M, et al.	4703406	ral squamous cell carcinoma (floor of the mout	11	18.18%
Grimm M, et al.	4703406	ral squamous cell carcinoma (floor of the mout	41	41.46%
Grimm M, et al.	4703406	ral squamous cell carcinoma (floor of the mout	64	43.75%
Grimm M, et al.	4703406	ral squamous cell carcinoma (floor of the mout	15	60.00%
Grimm M, et al.	4703406	ral squamous cell carcinoma (floor of the mout	8	37.50%
Grimm M, et al.	4703406	ral squamous cell carcinoma (floor of the mout	22	36.36%
Su XY, et al.	26191237	Thymoma	16	50.00%
Chikamoto A, et al.	28180987	Ductal adenocarcinoma of the pancreas	138	50.00%
Yen TC, et al.	14734665	Squamous cell carcinoma of the cervix	51	94.12%
Yen TC, et al.	14734665	Squamous cell carcinoma of the cervix	20	0.00%
Brinkhuizen T, et al.	25181405	Basal cell carcinoma of the skin	38	78.95%
Hirose Y,. Et al.	27218430	Squamous cell carcinoma of the esophagus	51	82.35%
Němejcová K, et al.	28381136	Endometrioid carcinoma of the ovary	184	86.96%
Němejcová K, et al.	28381136	Serous carcinoma of the ovary	29	100.00%
Němejcová K, et al.	28381136	Clear cell carcinoma of the ovary	17	100.00%
Kim BH, et al.	23510334	Papillary thyroid carcinoma	25	60.00%
Younes M, et al.	8640778	Squamous cell carcinoma of the skin	15	93.33%
Younes M, et al.	8640778	Squamous cell carcinoma of the skin	10	100.00%
Younes M, et al.	8640778	Basal cell carcinoma of the skin	15	86.67%
Kaira K, et al.	30077123	Adenocarcinoma of the lung	315	53.33%
Doki Y, et al.	15855926	Squamous cell carcinoma of the esophagus	80	43.75%
Westerterp M, et al.	17653575	Adenocarcinoma of the esophagus	18	55.56%
de SouzaLB, et al.	28299426	Pleomorphic adenoma of the parotid gland	20	90.00%
Kondo Y, et al.	21399882	ral squamous cell carcinoma (floor of the mout	107	98.13%
Thomas de Montprévil	26534878	Thymoma	11	90.91%
Thomas de Montprévil	26534878	Thymoma	6	83.33%
Thomas de Montprévil	26534878	Thymoma	11	90.91%
Thomas de Montprévil	26534878	Thymoma	25	96.00%
Thomas de Montprévil	26534878	Thymoma	5	60.00%
Thomas de Montprévil	26534878	Thymoma	17	100.00%
Schönberger J, et al.	12481939	Anaplastic thyroid carcinoma	5	100.00%
Schönberger J, et al.	12481939	Follicular thyroid carcinoma	20	65.00%
Schönberger J, et al.	12481939	Papillary thyroid carcinoma	20	100.00%
Nagamatsu A, et al.	20204293	Carcinosarcoma of the uterus	13	76.92%
Younes M, et al.	9816281	Adenocarcinoma of the colon	53	83.02%
Zhang X, et al.	23811806	ral squamous cell carcinoma (floor of the mout	36	88.89%
Kubo T, et al.	25193692	Osteosarcoma	37	32.43%
Üçer Ö	23661345	Adenocarcinoma of the lung	30	83.33%
Yokobori Y, et al.	25739639	ral squamous cell carcinoma (floor of the mout	27	92.59%
Kim YH, et al.	25053597	Lobular carcinoma of the breast	114	6.14%
