# Supplementary material for: Glucose-transporter 1 (GLUT1) as a prognostic biomarker: evidence from 14,966 human tumors across 134 cancer types
Source: BMC Cancer. 2026 Jan 10;26:127. doi: 10.1186/s12885-025-15527-5 (PMC12836948; doi:10.1186/s12885-025-15527-5)
Supplement: Supplementary file 3 — Supplementary Material 3. Supplementary Table 2. Gender and age of the patients, where available. N/A = Not assessed. [file 12885_2025_15527_MOESM3_ESM.pdf]

**Supplementary table 2.** Gender and age of the patients, where available. N/A = Not assessed.

| Tumor type | Gender (% of patients) | | Age at diagnosis, years (% of patients) | | | | |
|---|---|---|---|---|---|---|---|
| | male | female | ≤50 | 51-60 | 61-70 | 71-80 | >80 |
| Pilomatricoma | 55.9 | 44.1 | 88.6 | 8.6 | 2.9 | 0.0 | 0.0 |
| Basal cell carcinoma of the skin | 63.4 | 36.6 | 4.9 | 16.4 | 16.4 | 24.6 | 37.7 |
| Benign nevus | 31.0 | 69.0 | 69.0 | 10.3 | 13.8 | 3.4 | 3.4 |
| Squamous cell carcinoma of the skin | 64.8 | 35.2 | 2.1 | 11.3 | 16.3 | 29.8 | 40.4 |
| Malignant melanoma | 51.6 | 48.4 | 14.3 | 6.3 | 25.4 | 33.3 | 20.6 |
| Malignant melanoma lymph node metastasis | 54.7 | 45.3 | N/A | N/A | N/A | N/A | N/A |
| Merkel cell carcinoma | 56.3 | 43.8 | 2.1 | 4.2 | 14.6 | 35.4 | 43.8 |
| Squamous cell carcinoma of the larynx | 88.0 | 12.0 | 7.4 | 32.4 | 32.4 | 20.4 | 7.4 |
| Squamous cell carcinoma of the pharynx | 70.2 | 29.8 | 10.5 | 31.6 | 26.3 | 26.3 | 5.3 |
| Oral squamous cell carcinoma | 64.6 | 35.4 | 17.7 | 26.2 | 32.3 | 21.5 | 2.3 |
| Pleomorphic adenoma of the parotid gland | 40.0 | 60.0 | 50.0 | 14.0 | 20.0 | 10.0 | 6.0 |
| Warthin tumor of the parotid gland | 71.4 | 28.6 | 18.4 | 24.5 | 28.6 | 22.4 | 6.1 |
| Adenocarcinoma, (Papillary Cystadenocarcinoma) | N/A | N/A | N/A | N/A | N/A | N/A | N/A |
| Salivary duct carcinoma | N/A | N/A | N/A | N/A | N/A | N/A | N/A |
| Acinic cell carcinoma of the salivary gland | N/A | N/A | N/A | N/A | N/A | N/A | N/A |
| Adenocarcinoma NOS of the salivary gland | N/A | N/A | N/A | N/A | N/A | N/A | N/A |
| Adenoid cystic carcinoma of the salivary gland | N/A | N/A | N/A | N/A | N/A | N/A | N/A |
| Basal cell adenocarcinoma of the salivary gland | N/A | N/A | N/A | N/A | N/A | N/A | N/A |
| Basal cell adenoma of the salivary gland | 33.3 | 66.7 | 26.7 | 13.3 | 33.3 | 13.3 | 13.3 |
| Epithelial-myoepithelial carcinoma of the salivary gland | N/A | N/A | N/A | N/A | N/A | N/A | N/A |
| Mucoepidermoid carcinoma of the salivary gland | N/A | N/A | N/A | N/A | N/A | N/A | N/A |
| Myoepithelial carcinoma of the salivary gland | N/A | N/A | N/A | N/A | N/A | N/A | N/A |
| Myoepithelioma of the salivary gland | N/A | N/A | N/A | N/A | N/A | N/A | N/A |
| Oncocytic carcinoma of the salivary gland | N/A | N/A | N/A | N/A | N/A | N/A | N/A |
| Polymorphous adenocarcinoma, low grade, of the salivary gland | N/A | N/A | N/A | N/A | N/A | N/A | N/A |
| Pleomorphic adenoma of the salivary gland | N/A | N/A | N/A | N/A | N/A | N/A | N/A |
| Adenocarcinoma of the lung | 72.0 | 28.0 | 12.0 | 30.0 | 40.0 | 18.0 | 0.0 |
| Squamous cell carcinoma of the lung | 74.0 | 26.0 | 12.0 | 28.0 | 32.0 | 22.0 | 6.0 |
| Small cell carcinoma of the lung | 60.0 | 40.0 | 5.0 | 10.0 | 50.0 | 35.0 | 0.0 |
| Mesothelioma, epithelioid | 70.0 | 30.0 | 0.0 | 0.0 | 100.0 | 0.0 | 0.0 |
| Mesothelioma, biphasic | 84.2 | 15.8 | 4.1 | 8.2 | 20.4 | 53.1 | 14.3 |
| Thymoma | 55.2 | 44.8 | 24.1 | 10.3 | 37.9 | 17.2 | 10.3 |
| Lung, neuroendocrine tumor | 30.0 | 70.0 | 10.0 | 40.0 | 50.0 | 0.0 | 0.0 |
| Squamous cell carcinoma of the vagina | 12.3 | 87.7 | 17.6 | 21.6 | 23.0 | 28.4 | 9.5 |
| Squamous cell carcinoma of the vulva | 0.6 | 99.4 | 13.6 | 15.6 | 21.4 | 32.5 | 16.9 |
| Squamous cell carcinoma of the cervix | 0.8 | 99.2 | 45.4 | 23.1 | 19.2 | 6.2 | 6.2 |
| Adenosquamous carcinoma of the cervix | N/A | N/A | N/A | N/A | N/A | N/A | N/A |
| Adenocarcinoma of the cervix | 0.0 | 100.0 | 100.0 | 0.0 | 0.0 | 0.0 | 0.0 |
| Endometrioid endometrial carcinoma | 0.0 | 100.0 | 8.9 | 16.1 | 37.9 | 24.2 | 12.9 |
| Endometrial serous carcinoma | 0.0 | 100.0 | 7.7 | 11.5 | 32.7 | 26.9 | 21.2 |
| Carcinosarcoma of the uterus | 0.0 | 100.0 | 3.7 | 25.9 | 25.9 | 22.2 | 22.2 |
| Endometrial carcinoma, high grade, G3 | N/A | N/A | N/A | N/A | N/A | N/A | N/A |
| Endometrial clear cell carcinoma | 0.0 | 100.0 | N/A | N/A | N/A | N/A | N/A |
| Endometrioid carcinoma of the ovary | 0.0 | 100.0 | 32.1 | 32.1 | 19.0 | 13.1 | 3.6 |
| Serous carcinoma of the ovary | 0.0 | 100.0 | 16.2 | 29.1 | 24.3 | 25.0 | 5.4 |
| Mucinous carcinoma of the ovary | 0.0 | 100.0 | 30.4 | 30.4 | 16.1 | 8.9 | 14.3 |
| Clear cell carcinoma of the ovary | 0.0 | 100.0 | 18.2 | 22.7 | 27.3 | 27.3 | 4.5 |
| Carcinosarcoma of the ovary | 0.0 | 100.0 | 5.9 | 11.8 | 23.5 | 41.2 | 17.6 |
| Granulosa cell tumor of the ovary | 0.0 | 100.0 | 50.0 | 0.0 | 16.7 | 16.7 | 16.7 |
| Leydig cell tumor of the ovary | 0.0 | 100.0 | N/A | N/A | N/A | N/A | N/A |
| Sertoli cell tumor of the ovary | 0.0 | 100.0 | N/A | N/A | N/A | N/A | N/A |
| Sertoli Leydig cell tumor of the ovary | 0.0 | 100.0 | N/A | N/A | N/A | N/A | N/A |
| Steroid cell tumor of the ovary | 0.0 | 100.0 | N/A | N/A | N/A | N/A | N/A |
| Brenner tumor | 0.0 | 100.0 | 11.1 | 33.3 | 33.3 | 11.1 | 11.1 |
| Invasive breast carcinoma of no special type | 0.4 | 99.6 | 21.5 | 20.9 | 28.2 | 19.6 | 9.7 |
| Lobular carcinoma of the breast | 0.0 | 100.0 | 15.4 | 17.6 | 31.3 | 24.6 | 11.2 |
| Medullary carcinoma of the breast | 0.0 | 100.0 | 47.1 | 23.5 | 23.5 | 5.9 | 0.0 |
| Tubular carcinoma of the breast | 0.0 | 100.0 | 41.4 | 24.1 | 24.1 | 0.0 | 10.3 |
| Mucinous carcinoma of the breast | 1.5 | 98.5 | 12.3 | 4.6 | 21.5 | 36.9 | 24.6 |
| Phyllodes tumor of the breast | 0.0 | 100.0 | 57.1 | 24.5 | 16.3 | 2.0 | 0.0 |
| Adenomatous polyp, low-grade dysplasia | 60.0 | 40.0 | 10.0 | 10.0 | 30.0 | 32.0 | 18.0 |
| Adenomatous polyp, high-grade dysplasia | 60.0 | 40.0 | 2.0 | 8.0 | 34.0 | 36.0 | 20.0 |
| Adenocarcinoma of the colon | 56.0 | 44.0 | 7.6 | 16.7 | 19.7 | 40.9 | 15.2 |
| Gastric adenocarcinoma, diffuse type | 50.8 | 49.2 | 16.0 | 16.7 | 25.0 | 28.2 | 14.1 |
| Gastric adenocarcinoma, intestinal type | 64.1 | 35.9 | 3.6 | 12.7 | 18.2 | 37.0 | 28.5 |
| Gastric adenocarcinoma, mixed type | 69.4 | 30.6 | N/A | N/A | N/A | N/A | N/A |
| Adenocarcinoma of the esophagus | 79.7 | 20.3 | 11.3 | 22.6 | 39.8 | 24.1 | 2.3 |
| Squamous cell carcinoma of the esophagus | 72.0 | 28.0 | 12.8 | 34.4 | 30.4 | 19.2 | 3.2 |
| Squamous cell carcinoma of the anal canal | 42.9 | 57.1 | 26.4 | 28.6 | 20.9 | 18.7 | 5.5 |
| Cholangiocarcinoma | 44.5 | 55.5 | 12.1 | 17.2 | 39.7 | 29.3 | 1.7 |
| Gallbladder adenocarcinoma | 30.6 | 69.4 | 8.2 | 14.3 | 26.5 | 36.7 | 14.3 |
| Gallbladder Klatskin tumor | 64.3 | 35.7 | 14.3 | 14.3 | 28.6 | 35.7 | 7.1 |
| Hepatocellular carcinoma | 71.3 | 28.7 | 9.4 | 19.4 | 33.2 | 31.0 | 7.1 |
| Ductal adenocarcinoma of the pancreas | 51.1 | 48.9 | 9.8 | 18.5 | 32.4 | 33.5 | 5.8 |
| Pancreatic/Ampullary adenocarcinoma | 63.1 | 36.9 | 7.5 | 13.4 | 29.9 | 38.8 | 10.4 |
| Acinar cell carcinoma of the pancreas | 60.0 | 40.0 | 10.0 | 10.0 | 40.0 | 40.0 | 0.0 |
| Gastrointestinal stromal tumor | 48.4 | 51.6 | 4.8 | 19.4 | 30.6 | 37.1 | 8.1 |
| Appendix, neuroendocrine tumor (NET) | 33.3 | 66.7 | 0.0 | 100.0 | 0.0 | 0.0 | 0.0 |
| Colorectal, neuroendocrine tumor (NET) | 0.0 | 100.0 | N/A | N/A | N/A | N/A | N/A |
| Ileum, neuroendocrine tumor (NET) | 75.0 | 25.0 | 25.0 | 0.0 | 25.0 | 25.0 | 25.0 |
| Pancreas, neuroendocrine tumor (NET) | 54.2 | 45.8 | 34.0 | 21.3 | 21.3 | 23.4 | 0.0 |
| Colorectal, neuroendocrine carcinoma (NEC) | 100.0 | 0.0 | 0.0 | 0.0 | 50.0 | 50.0 | 0.0 |
| Ileum, neuroendocrine carcinoma (NEC) | 37.5 | 62.5 | 0.0 | 25.0 | 50.0 | 12.5 | 12.5 |
| Pancreas, neuroendocrine carcinoma (NEC) | 0.0 | 100.0 | 0.0 | 0.0 | 0.0 | 100.0 | 0.0 |
| Non-invasive papillary urothelial carcinoma, pTa G2 low grade | 81.8 | 18.2 | N/A | N/A | N/A | N/A | N/A |
| Non-invasive papillary urothelial carcinoma, pTa G2 high grade | 80.5 | 19.5 | N/A | N/A | N/A | N/A | N/A |
| Non-invasive papillary urothelial carcinoma, pTa G3 | 85.3 | 14.7 | N/A | N/A | N/A | N/A | N/A |
| Urothelial carcinoma, pT2-4 G3 | 75.0 | 25.0 | 4.0 | 18.0 | 24.0 | 42.0 | 12.0 |
| Squamous cell carcinoma of the bladder | 36.4 | 63.6 | N/A | N/A | N/A | N/A | N/A |
| Small cell neuroendocrine carcinoma of the bladder | 86.4 | 13.6 | 0.0 | 11.1 | 44.4 | 38.9 | 5.6 |
| Urothelial carcinoma of the kidney pelvis | 62.9 | 37.1 | N/A | N/A | N/A | N/A | N/A |
| Clear cell renal cell carcinoma | 68.7 | 31.3 | 9.8 | 19.0 | 32.9 | 27.5 | 10.8 |
| Papillary renal cell carcinoma | 76.2 | 23.8 | 12.3 | 22.7 | 30.7 | 28.2 | 6.1 |
| Clear cell (tubulo) papillary renal cell carcinoma | 66.7 | 33.3 | 20.0 | 20.0 | 40.0 | 20.0 | 0.0 |
| Chromophobe renal cell carcinoma | 63.2 | 36.8 | 16.9 | 30.3 | 23.6 | 23.6 | 5.6 |
| Oncocytoma of the kidney | 60.6 | 39.4 | 8.5 | 11.5 | 32.3 | 42.3 | 5.4 |
| Adenocarcinoma of the prostate, Gleason 3+3 | 100.0 | 0.0 | 8.4 | 37.3 | 48.2 | 6.0 | 0.0 |
| Adenocarcinoma of the prostate, Gleason 4+4 | 100.0 | 0.0 | 1.3 | 18.8 | 50.0 | 30.0 | 0.0 |
| Adenocarcinoma of the prostate, Gleason 5+5 | 100.0 | 0.0 | 3.5 | 12.9 | 50.6 | 32.9 | 0.0 |
| Adenocarcinoma of the prostate (recurrence) | 100.0 | 0.0 | N/A | N/A | N/A | N/A | N/A |
| Small cell neuroendocrine carcinoma of the prostate | 100.0 | 0.0 | 0.0 | 11.8 | 35.3 | 47.1 | 5.9 |
| Seminoma | 99.1 | 0.9 | 86.1 | 8.3 | 4.6 | 0.9 | 0.0 |
| Embryonal carcinoma of the testis | 100.0 | 0.0 | 98.1 | 1.9 | 0.0 | 0.0 | 0.0 |
| Leydig cell tumor of the testis | 100.0 | 0.0 | 0.0 | 100.0 | 0.0 | 0.0 | 0.0 |
| Sertoli cell tumor of the testis | 100.0 | 0.0 | N/A | N/A | N/A | N/A | N/A |
| Sex cord stromal tumor of the testis | 100.0 | 0.0 | N/A | N/A | N/A | N/A | N/A |
| Spermatocytic tumor of the testis | 100.0 | 0.0 | N/A | N/A | N/A | N/A | N/A |
| Yolk sac tumor | 100.0 | 0.0 | 98.1 | 0.0 | 1.9 | 0.0 | 0.0 |
| Teratoma | 30.2 | 69.8 | 88.7 | 3.8 | 1.9 | 5.7 | 0.0 |
| Squamous cell carcinoma of the penis | 100.0 | 0.0 | 5.6 | 13.3 | 33.3 | 30.0 | 17.8 |
| Adenoma of the thyroid gland | 30.3 | 69.7 | 62.0 | 12.0 | 18.0 | 6.0 | 2.0 |
| Papillary thyroid carcinoma | 28.5 | 71.5 | 60.0 | 24.0 | 14.0 | 2.0 | 0.0 |
| Follicular thyroid carcinoma | 29.6 | 70.4 | 30.2 | 23.3 | 27.9 | 16.3 | 2.3 |
| Medullary thyroid carcinoma | 35.1 | 64.9 | 35.2 | 25.9 | 22.2 | 16.7 | 0.0 |
| Parathyroid gland adenoma | 27.9 | 72.1 | N/A | N/A | N/A | N/A | N/A |
| Anaplastic thyroid carcinoma | 37.8 | 62.2 | 0.0 | 19.2 | 42.3 | 26.9 | 11.5 |
| Adrenal cortical adenoma | 33.3 | 66.7 | 41.7 | 20.8 | 25.0 | 12.5 | 0.0 |
| Adrenal cortical carcinoma | 48.1 | 51.9 | 55.6 | 18.5 | 18.5 | 7.4 | 0.0 |
| Pheochromocytoma | 64.0 | 36.0 | 42.0 | 28.0 | 20.0 | 10.0 | 0.0 |
| Hodgkin's lymphoma | 66.7 | 33.3 | 77.8 | 4.4 | 8.9 | 8.9 | 0.0 |
| Tendosynovial giant cell tumor | 48.9 | 51.1 | 62.2 | 22.2 | 8.9 | 6.7 | 0.0 |
| Granular cell tumor | 35.8 | 64.2 | 50.0 | 30.0 | 13.3 | 6.7 | 0.0 |
| Leiomyosarcoma | 22.9 | 77.1 | 0.0 | 30.0 | 30.0 | 40.0 | 0.0 |
| Liposarcoma | 60.0 | 40.0 | 27.4 | 12.9 | 27.4 | 27.4 | 4.8 |
| Malignant peripheral nerve sheath tumor | 53.3 | 46.7 | 0.0 | 0.0 | 50.0 | 50.0 | 0.0 |
| Myofibrosarcoma | 65.4 | 34.6 | N/A | N/A | N/A | N/A | N/A |
| Angiosarcoma | 61.6 | 38.4 | 18.8 | 9.4 | 18.8 | 25.0 | 28.1 |
| Angiomyolipoma | 25.6 | 74.4 | N/A | N/A | N/A | N/A | N/A |
| Dermatofibrosarcoma protuberans | 42.9 | 57.1 | N/A | N/A | N/A | N/A | N/A |
| Ganglioneuroma | 50.0 | 50.0 | N/A | N/A | N/A | N/A | N/A |
| Kaposi sarcoma | 50.0 | 50.0 | N/A | N/A | N/A | N/A | N/A |
| Neurofibroma | 45.3 | 54.7 | N/A | N/A | N/A | N/A | N/A |
| Sarcoma, not otherwise specified | 51.4 | 48.6 | N/A | N/A | N/A | N/A | N/A |
| Paraganglioma | 39.0 | 61.0 | N/A | N/A | N/A | N/A | N/A |
| Ewing sarcoma | 69.6 | 30.4 | N/A | N/A | N/A | N/A | N/A |
| Rhabdomyosarcoma | 71.4 | 28.6 | 0.0 | 100.0 | 0.0 | 0.0 | 0.0 |
| Schwannoma | 46.7 | 53.3 | 0.0 | 0.0 | 100.0 | 0.0 | 0.0 |
| Synovial sarcoma | 41.7 | 58.3 | N/A | N/A | N/A | N/A | N/A |
| Osteosarcoma | 52.3 | 47.7 | 53.8 | 7.7 | 15.4 | 19.2 | 3.8 |
| Chondrosarcoma | 52.5 | 47.5 | 44.4 | 7.4 | 3.7 | 40.7 | 3.7 |
| Rhabdoid tumor | 60.0 | 40.0 | N/A | N/A | N/A | N/A | N/A |
