# Supplementary material for: Glucose-transporter 1 (GLUT1) as a prognostic biomarker: evidence from 14,966 human tumors across 134 cancer types
Source: BMC Cancer. 2026 Jan 10;26:127. doi: 10.1186/s12885-025-15527-5 (PMC12836948; doi:10.1186/s12885-025-15527-5)
Supplement: Supplementary file 4 — Supplementary Material 4. Supplementary Table 3. Multivariate analysis of GLUT1 immunostaining versus tumor stage andgrade in clear cell renal cell carcinomas. [file 12885_2025_15527_MOESM4_ESM.pdf]

Supplementary Table 3: Multivariate analysis of GLUT1 immunostaining versus tumor stage and grade in clear cell renal cell carcinomas.

End point	Parameter		HR	95% CI	P
Recurrence free survival	pT	pT2 vs pT1	1.36	0.4 - 4.7	*<0.001*
		pT3 vs pT1	4.5	2.3 - 8.8	
	pN	pN1 vs pN0	2.95	1.2 - 7.3	*0.0129*
		pN2 vs pN0	3	1.3 - 7.0	
	GLUT1	weak vs negative	0.4	0.1 - 1.6	*0.1253*
		mod. vs negative	0.9	0.2 - 3.6	
		str. vs negative	1.1	0.3 - 3.6	
Overall survival	pT	pT2 vs pT1	0.9	0.3 - 3.3	*0.0001*
		pT3 vs pT1	3.8	1.8 - 7.7	
	pN	pN1 vs pN0	2.1	0.7 - 5.9	*0.0034*
		pN2 vs pN0	1.9	0.6 - 6.1	
	GLUT1	weak vs negative	0.5	0.2 - 1.9	*0.0624*
		mod. vs negative	1.0	0.3 - 3.6	
		str. vs negative	1.7	0.6 - 4.3	
